# Native functions of short tandem repeats

**DOI:** 10.7554/eLife.84043

**Published:** 2023-03-20

**Authors:** Shannon E Wright, Peter K Todd

**Affiliations:** 1 Department of Neurology, University of Michigan–Ann Arbor Ann Arbor United States; 2 Neuroscience Graduate Program, University of Michigan–Ann Arbor Ann Arbor United States; 3 Department of Neuroscience, Picower Institute Cambridge United States; 4 VA Ann Arbor Healthcare System Ann Arbor United States; Chinese Academy of Sciences China; Stanford University United States

**Keywords:** neurodegeneration, genomic instability, Fragile X, gene expression, disease mechanism, autism

## Abstract

Over a third of the human genome is comprised of repetitive sequences, including more than a million short tandem repeats (STRs). While studies of the pathologic consequences of repeat expansions that cause syndromic human diseases are extensive, the potential native functions of STRs are often ignored. Here, we summarize a growing body of research into the normal biological functions for repetitive elements across the genome, with a particular focus on the roles of STRs in regulating gene expression. We propose reconceptualizing the pathogenic consequences of repeat expansions as aberrancies in normal gene regulation. From this altered viewpoint, we predict that future work will reveal broader roles for STRs in neuronal function and as risk alleles for more common human neurological diseases.

## Introduction

At least a third of the human genome is comprised of repetitive sequences (de Koning et al., 2011; Gemmell, 2021; Britten and Kohne, 1968). Some of the first genomic repetitive elements were discovered in association with disease. As a result, pathogenic roles of repeats were well studied, while potential native functions of these repeats were largely dismissed. However, the conservation of genomic repeats among different eukaryotic species (Eichler et al., 1995; Sulovari et al., 2019; Liquori et al., 2003) and their high polymorphism rates compared to other types of genetic variations (Willems et al., 2016) suggests that repeats may have important biological functions in addition to the pathogenic ones. A growing body of research has revealed complex biological and evolutionary functions for repeats across the genome. Here, we summarize the important functions of one type of genomic repeat, short (2–6 base pair) tandem repeats (STRs), in DNA, RNA, and as proteins. We then reframe STR toxicity observed in repeat expansion disorders (REDs) as an aberrancy of native STR functions, rather than as solely a emergent property disconnected from the native repeat. Finally, we discuss how this alternative view of STR toxicity can improve our understanding of roles of STRs in neuronal function and human health.

### A brief history of repetitive DNA

Repetitive elements in DNA were first discovered by Barbara McClintock, who observed the presence of ‘controlling elements’ randomly dispersed throughout the maize (Zea mays) genome (Comfort, 2001; Ravindran, 2012; McClintock, 1950). These interspersed repeats, which would come to be known as transposable elements (TEs), use flanking repetitive sequences to ‘jump’ around to different locations in the genome, often resulting in duplications of genetic material.

In contrast to interspersed repeats, tandem repeats (TRs) are regions in which repeating units lie in parallel (or in tandem) and are classified by size of the repeating unit as satellites (>60 base pairs), minisatellites (10–60 base pairs), or microsatellites (<9 base pairs). Short (2–6 base pair) tandem repeats (STRs) comprise between 1% and 3% of the human genome (Gymrek et al., 2016; Wyner et al., 2020; Lander et al., 2001). In the early 1990s, a series of STR expansions were causally linked with human diseases, including spinobulbar muscular atrophy (La Spada et al., 1992), Fragile X Syndrome (Fu et al., 1991; Heitz et al., 1991; Oberlé et al., 1991; Verkerk et al., 1991; Yu et al., 1991), Huntington’s disease (MacDonald et al., 1993), and myotonic dystrophy (Brook et al., 1992; Buxton et al., 1992; Harley et al., 1992; Aslanidis et al., 1992; Fu et al., 1992; Mahadevan et al., 1992). As such, much of the research on STRs to date has centered on the mechanisms by which repeat expansions trigger neuronal toxicity. We will use the Fragile X locus as an exemplar of this now extensive body of literature, which is reviewed in more detail elsewhere (Malik et al., 2021a; Hagerman et al., 2017; Hagerman and Hagerman, 2015; Jacquemont et al., 2003; Glineburg et al., 2018), as it helps us understand how STRs might function normally in the absence of expansion.

#### Fragile X-associated disorders: the discovery of pathogenic short tandem repeats

Fragile X Syndrome (FXS), the most common monogenic form of intellectual disability, was one of the first genetic diseases linked to an STR expansion (Fu et al., 1991; Heitz et al., 1991; Oberlé et al., 1991; Verkerk et al., 1991; Yu et al., 1991). In 1943, Julia Bell and James Purdon Martin described an X-linked intellectual disability primarily affecting people assigned male at birth, that could be inherited from a carrier female parent or affected male parent (Martin and Bell, 1943). Karyotypes of affected individuals show a folate-sensitive fragile site on the X chromosome, which causes the chromosome to bend or break at one arm (Lubs, 1969; Proops and Webb, 1981; Chudley and Hagerman, 1987; Hagerman et al., 1986). The fragile site associated with FXS is located at the Fragile X messenger ribonucleoprotein 1 (*FMR1*) gene, which contains a large CGG repeat in the 5’ UTR of affected individuals (>200 repeats) (Fu et al., 1991; Heitz et al., 1991; Oberlé et al., 1991; Verkerk et al., 1991; Yu et al., 1991). In addition to intellectual disability, FXS patients commonly present with hyperactivity, anxiety, and seizures (Hagerman et al., 2017; Chudley and Hagerman, 1987; Hagerman and Hagerman, 2002a). Other chromosomal fragile sites also contain STRs, some of which are linked to other diseases (Glover, 2006; Debacker and Kooy, 2007; Schwartz et al., 2006). For example, Fragile XE syndrome (FRAXE), caused by a CGG repeat expansion in the *FMR2* gene (Knight et al., 1993; Gu et al., 1996; Gecz et al., 1996), manifests in an X-linked intellectual disability similar to FXS (Mulley et al., 1995; Gecz, 2000).

While studying pedigrees of Fragile X families, Stephanie Sherman and colleagues observed incomplete penetrance of mental impairment, affecting only 79% of males and 35% of females (Sherman et al., 1984; Sherman et al., 1985). This ‘Sherman paradox’ suggested a generational risk factor in Fragile X mental impairment, as unaffected ‘normal transmitting’ males (NTMs) passed on a mutant allele to unaffected female children, with disease manifestation in affected (predominantly) male grandchildren. Subsequent studies of CGG repeat length variation found that individuals from non-Fragile X families have 6–54 CGG repeats, while some unaffected individuals in Fragile X families have 55–200 repeats, a ‘pre-mutation’ associated with increased risk of further repeat expansion during oogenesis (Fu et al., 1991).

Subsequent work with Fragile X families revealed that Fragile X premutation expansion carriers often manifest clinically distinct disorders that are caused by the CGG repeat. Fragile X-associated tremor/ataxia syndrome (FXTAS) is an age-linked neurodegenerative disorder characterized by progressive intention tremor and ataxia, parkinsonism, and cognitive decline (Hagerman and Hagerman, 2015; Jacquemont et al., 2003; Hagerman and Hagerman, 2004; Hagerman et al., 2001; Hagerman and Hagerman, 2002b; Leehey et al., 2003; Brunberg et al., 2002). As an X-linked disorder, FXTAS primarily affects people assigned male at birth. People with two X chromosomes may develop FXTAS, but are also at risk for developing Fragile X-associated premature ovarian insufficiency (FXPOI), a disorder characterized by absent or irregular menstrual cycles, early onset of menopause, and fertility issues (Hagerman and Hagerman, 2002a; Allingham-Hawkins et al., 1999; Murray et al., 2000a; Murray et al., 2000b). As ‘premutation disorders’, FXTAS and FXPOI are thought to share similar molecular mechanisms by which the premutation CGG repeat expansion causes cytotoxicity and dysfunction.

More than 50 REDs discovered to date show common mechanisms of molecular pathology (Malik et al., 2021a; Glineburg et al., 2018; Paulson, 2018; Rodriguez and Todd, 2019). FXS, FXTAS, and FXPOI, collectively referred to as Fragile X-associated disorders, are revisited throughout this review to exemplify the mechanisms by which STRs can cause cellular dysfunction and toxicity. However, the often-stereotyped manifestations of REDs, in addition to the abundance of repetitive elements throughout the genome, suggests that STRs could have native functions which become aberrant in the setting of repeat expansions. We will focus most of the rest of the review on this supposition.

### Native STR functions

While overshadowed by disease-centered research, scientists have investigated functional consequences of repeat polymorphisms for decades. Studies of individual or small groups of genes showed phenotypic consequences of repeat length variation on flocculation and cell adhesion in yeast (Voynov et al., 2006; Levdansky et al., 2007; Verstrepen et al., 2005), limb and skull morphology in dogs (Fondon and Garner, 2004) and on behavioral traits in voles (Hammock and Young, 2005). Recent advances in sequencing technology and STR-conscious alignment techniques now permit the detection and characterization of thousands of new STRs and their variation across the human genome, and have enabled genome-wide study of the effect of repeat length polymorphisms on gene expression (Willems et al., 2016; Payseur et al., 2011; McIver et al., 2011; O’Dushlaine and Shields, 2008; McIver et al., 2013; Willems et al., 2014; Mallick et al., 2016). As thousands of single-nucleotide polymorphisms (SNPs) have been linked with disease risk in Genome Wide Association Studies (GWAS; Tam et al., 2019; Uffelmann et al., 2021), ongoing studies of human genomes aim to link variation in STR length to phenotypic outcomes (Gymrek et al., 2016; Fotsing et al., 2019; Mitra et al., 2021). In homage to the expression quantitative trait loci (eQTL) identified in traditional GWAS (Tam et al., 2019; Uffelmann et al., 2021), STRs associated with differences in expression of nearby genes are called eSTRs. In the following sections, we will first discuss evidence for evolutionary constraint on STRs linked to evolution across phylogeny and in humans. We will then showcase the mechanisms by which variation in STR length affects gene expression.

#### Repetitive DNA regulates transcription

**Figure 1.**
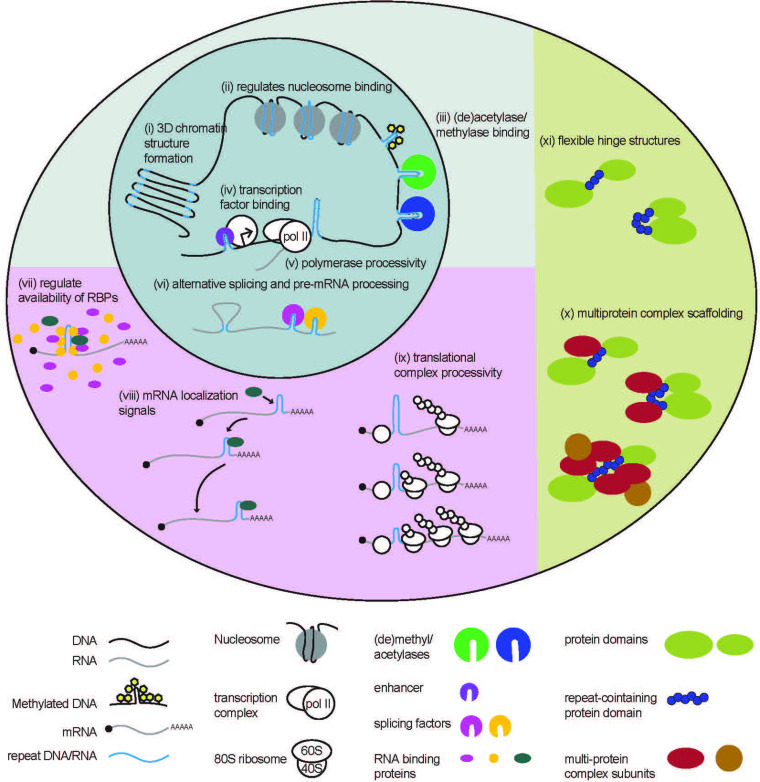
Native functions of genomic repeats. Repeats in DNA influence larger 3D chromatin structures, regulate binding of nucleosomes and (de)acetylases and (de)methylases. They also influence transcription factor binding and polymerase processivity to affect downstream RNA production. Repeats in RNA can affect pre-mRNA processing such as alternative splicing and can affect RNA binding protein function through direct or indirect sequestration. Repeats in 3’ UTRs serve as localization signals, directing mRNA transport. Repeats in 5’ UTRs regulate translational output by impeding ribosome processivity. Repeating units in proteins can provide structural flexibility within a protein or serve as binding sites for the formation of multi-protein complexes.

Repetitive elements can impact the transcription of neighboring genes or the genes in which they reside by regulating chromatin structure and epigenetic markers. A role for repetitive DNA in facilitating 3D folding of the genome was first observed with TE-dependent formation of chromatin loops across multiple species, including yeast, *Drosophila*, and mammals (Figure 1(i); Cournac et al., 2016; Bourque, 2009; Lu et al., 2021). Contact maps generated using chromosome conformation capture (Hi-C) show high co-localization of repetitive elements in nuclear space in humans, mice, and *Drosophila*, demonstrating a structural function of repetitive DNA (Cournac et al., 2016). The enrichment of transcription factor binding sites in proximity to spatially associated repeats suggests that repeat-mediated 3D DNA packaging may allow for context-dependent co-transcription of linearly remote genes (Cournac et al., 2016).

STRs play critical roles in maintaining chromatin structure (Nikumbh and Pfeifer, 2017; Sun et al., 2018; Volle and Delaney, 2012). For example, short CAG/CTG tracts avidly incorporate nucleosomes (Figure 1 (ii)), which are a basic subunit of chromatin packaging (Volle and Delaney, 2012). Nucleosome position varies with differences in STR length and flanking sequence context (Volle and Delaney, 2012), influencing chromatin structure and transcription of nearby genes. Other STRs, including CGG repeats, have the opposite effect and exclude nucleosomes in their native states, creating more open chromatin states near the transcription start sites of genes that favor local transcription (Wang et al., 1996; Wang, 2007). This feature may underlie the enrichment of CGG repeats in promoters and 5’UTRs (Uesaka et al., 2014).

STRs can also influence chromatin structure by modulating DNA methylation. Some STRs are prone to methylation, which can lead to gene silencing and the absence of transcription (Quilez et al., 2016; Garg et al., 2021; Pappalardo and Barra, 2021). One common example of repeat-mediated gene silencing, CpG islands are repeating di-nucleotide CpG sequences ranging from around 500–3000 base pairs and are located in ~40% of gene promoters across mammalian genomes (Deaton and Bird, 2011; Janitz and Janitz, 2011; Thomson et al., 2010; Clouaire et al., 2012; Blackledge et al., 2013). STRs are commonly located near CpG islands (Sun et al., 2018), and may influence their methylation states (Figure 1 (iii)). Moreover, other STRs can contain CpGs within their repetitive sequence that can undergo methylation (Bolton et al., 2013).

A genome-wide study in yeast (Vinces et al., 2009) estimates as many as 25% of promoters contain tandem repeats (TRs). Generally, expression of genes with TRs in their promoters increased with increasing repeat size. TRs in promoters may increase gene expression by increasing transcription factor binding (Figure 1 (iv)), blocking or reducing nucleosome density, or in the case of AT-rich repeats, by facilitating DNA melting (Vinces et al., 2009). However, stable secondary structures formed by TRs can also inhibit transcription by impeding procession and access of transcriptional machinery (Figure 1 (v); Grabczyk and Fishman, 1995; Belotserkovskii et al., 2010; Usdin and Woodford, 1995). For example, the evolutionarily conserved THO complex is recruited to actively transcribed genes (Kim et al., 2004; Abruzzi et al., 2004; Strässer et al., 2002), and facilitates elongation of RNA polymerase (Fan et al., 1996; Prado et al., 1997; Fan et al., 2001; Jimeno et al., 2002; Chávez et al., 2000) through super-helical structures formed by long GC-rich TRs (Voynov et al., 2006; Chávez et al., 2001). Yeast strains with mutations to THO complex subunits exhibited lower levels of TR-containing FLO11 mRNA. Reduced FLO11 mRNA coincided with an accumulation of RNA polymerase at the beginning of the gene. Removal of the TR or overexpression of topoisomerase I to enhance unwinding of the structured DNA, rescued the reduction in FLO11 mRNA in THO complex mutants (Voynov et al., 2006).

As in yeast, human STRs can either enhance and inhibit transcription of associated genes dependent on their sequence and locations, and also affect gene expression via changes in gene methylation and chromatin structure (Gymrek et al., 2016; Fotsing et al., 2019; Quilez et al., 2016; Garg et al., 2021; Jakubosky et al., 2020). Together, these studies demonstrate numerous mechanisms by which TRs can enhance or inhibit gene expression.

#### STRs in RNA regulate pre-mRNA processing and RNA localization

Transcribed repetitive elements regulate numerous aspects of RNA biology. STRs in RNAs form complex higher order structures, including G-quadruplexes and hairpins (Krzyzosiak et al., 2012; Sobczak et al., 2003; Malgowska et al., 2014; Sobczak et al., 2010), which are thought to exert broad influence over pre-mRNA splicing (Figure 1 (vi)) (Muro et al., 1999; Tu et al., 2000; Black, 2003; Solnick and Lee, 1987). An analysis of human introns found that sites of alternative splicing are enriched for STRs (Lian and Garner, 2005). STRs can facilitate alternative splicing by complementary pairing of intronic repeats, bringing exonic regions into close proximity (Lian and Garner, 2005). Structure-forming STRs can inhibit or enhance alternative splicing by blocking or facilitating the recruitment of splicing factors, respectively (Lian and Garner, 2005). For example, alternative splicing of the EIIIB exon in the well-conserved fibronectin gene is regulated by an intronic TGCATG repeat (Huh and Hynes, 1994; Lim and Sharp, 1998). Contractions in this STR reduce EIIIB exon inclusion, while overexpression of a specific splicing factor, SRp40, stimulates inclusion. While the TGCATG repeat differs from SRp40’s consensus binding site, it can form a strong hairpin structure, which is a key feature of SRp40 binding site motifs (Tacke et al., 1997). This suggests that the TGCATG repeat may modulate alternative splicing by recruiting the SRp40 splicing factor to the intron/exon boundary (Lim and Sharp, 1998).

Some STRs in RNA regulate splicing in trans, by binding to and sequestering splicing factors and blocking their functions (Figure 1 (vii)). A recent study identified a group of novel long non-coding RNAs (lncRNAs) with multiple predicted RNA binding motifs (Yap et al., 2018), a subset of which contained long stretches of STRs (‘strRNAs’). One strRNA called the pyrimidine-rich noncoding transcript (PNCTR) contains numerous stretches of (TC)_n_ repeats, avidly binds to the polypyrimidine tract-binding protein (PTBP1) in cells, and negatively regulates PTBP1-mediated splicing (Yap et al., 2018). As such, PNCTR overexpression was sufficient to trigger mis-splicing of PTBP1 targets and trigger programmed cell death (Yap et al., 2018). In this way, STRs in RNA can regulate the global availability of other RNA-binding proteins (RBPs) with other functions, exerting profound control over numerous aspects of cell biology.

STRs in 3’ UTRs can also serve as RNA localization signals, and via interactions with RBPs, facilitate the transport of RNAs to specified cellular compartments (Figure 1 (viii)). A program called REPFIND was developed to analyze 3’ UTRs of localized mRNAs in *Xenopus* oocytes and identified various CAC-containing repeat motifs that serve as localization elements (Betley et al., 2002). Mutating these CAC-containing repeats was sufficient to abolish normal RNA localization. CAC-containing repeats were also found in zebrafish and human 3’ UTRs of transcripts that are known to be specifically localized within cells, suggesting that CAC-containing repeats are conserved localization elements in chordates (Betley et al., 2002). REPFIND was subsequently used to generate a database of repeating motifs in 3’ UTRs of mammalian genes from the Mammalian Gene Collection (MGC) that revealed hundreds of human genes containing short CAC- and CAG-rich repeats in their 3’ UTRs (Lim and Sharp, 1998). Intriguingly, these elements facilitate RNA localization to neurites in rat hippocampal neurons (Andken et al., 2007).

#### Repetitive RNA regulates translation

STRs located in 5’ UTRs and coding regions impact mRNA translational efficiency. GC-rich STRs form stable RNA structures (Krzyzosiak et al., 2012; Sobczak et al., 2003; Malgowska et al., 2014; Sobczak et al., 2010), which can impede the processivity of scanning translational complexes (Figure 1 (ix)) (Kozak, 1980; Kozak, 1986; Kudla et al., 2009; Tuller et al., 2011; Ding et al., 2012; Bentele et al., 2013; Weinberg et al., 2016). For example, a native GGN repeat in the 5’ UTR of the potassium 2-pore domain leak channel Task3 mRNA forms a G-quadruplex structure in vivo (Maltby et al., 2020). This G-quadruplex is inhibitory to translation of Task3 mRNA, but can be overcome by DHX36 helicase activity to improve ribosome processivity through the stable structure (Maltby et al., 2020).

Indeed, libraries of synthetic (Millette et al., 2022) and naturally occurring (Li et al., 2017; Niederer et al., 2022) hairpin sequences placed within 5’ UTRs can be used to precisely control translational transgene output, with potential implications for gene therapy dosing. These studies show how single unit variations in STRs can precisely modulate protein expression, generally permitting more and faster translation of mRNAs with smaller STRs, and less and slower translation of mRNAs with larger STRs.

#### Repeats in proteins facilitate multi-protein complex formation and structural flexibility

Eukaryotic proteins are more likely to have repeats than prokaryotic proteins, and proteins containing repeats are often unique to eukaryotes and eukaryotic functions (Marcotte et al., 1999). There are numerous long repeating motifs in proteins (>20 amino acids/repeat) with loose homology between repeats, that form complex tertiary structures (Andrade et al., 2001). These protein repeat domains are characterized by the structures they form, as all-β (i.e. β-propellers, β-trefoils), all-α (i.e. HEAT and tetratricopeptide repeats (TPRs)), or mixed α/β (i.e. leucine-rich repeats, ankyrin repeat; Andrade et al., 2001). Although their specific functions vary, protein repeat domains typically serve as binding sites, and are thought to have evolved in eukaryotes to aide in the formation of multi-protein complexes with advanced cellular functions (Andrade et al., 2001; Kajava, 2012; Sharma and Pandey, 2015).

STRs translated into proteins, are thought to have similar functions as these larger repeat-based protein domains, serving as sites for protein-protein interactions (Figure 1 (x); Schaefer et al., 2012; Faux, 2012). CAG repeats are enriched in coding regions and are most frequently found in the polyglutamine (polyQ) reading frame, suggesting that polyQ stretches in proteins have a native function (Schaefer et al., 2012). PolyQ stretches are enriched in proteins that are components of multi-protein complexes, and have functions in transcriptional control, phosphatidylinositol (PI) signaling, protein degradation, and chromatin remodeling. Evolutionary sequence comparison reveals that the location of polyQs within a protein is not always conserved (Schaefer et al., 2012). This suggests that polyQ stretches have evolved multiple times, and don’t directly confer a protein’s function, but rather modulate the protein-protein interactions necessary for those functions (Schaefer et al., 2012; Orr, 2012). Other CG-containing STRs (i.e. CUGs and CGGs) show similar patterns of overrepresentation in coding regions (Schaefer et al., 2012), and likely serve similar complex-scaffolding functions (Nasrallah et al., 2012).

STRs when translated into proteins can be critical for proper protein folding. For example, translation of a CAG repeat in the huntingtin gene (*HTT*) produces a polyQ tract in the HTT protein which serves as a flexible hinge, allowing the neighboring domains to fold into close proximity (Figure 1 (xi)) (Caron et al., 2013). HTT protein structure is altered with repeat expansion, demonstrating the importance of the flexibility conferred by this STR (Caron et al., 2013).

### Pathogenic consequences of STRs: A Fragile X case study

**Figure 2.**
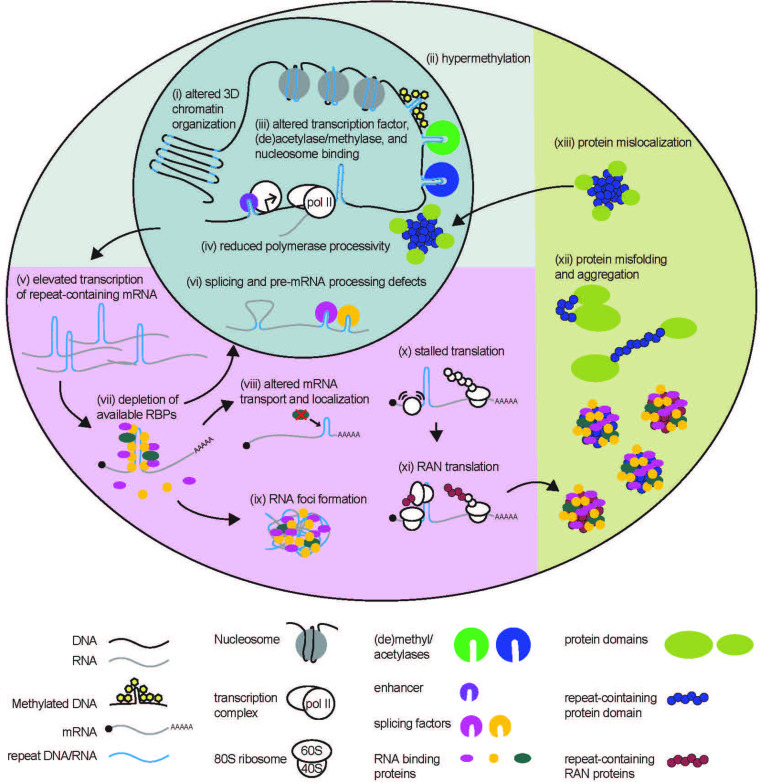
STR-associated toxicity in Repeat Expansion Disorders. Repeat expansions can alter global 3D chromatin structure, and influence transcription via blocking or enhancing binding of nucleosomes, (de)acetylases, (de)methylases, and transcription factors. Expanded repeats may also impede polymerase processivity. In some cases, elevated transcription of repeat expansion RNA can lead to depletion of RNA-binding proteins. Depletion of these proteins can impact many processes to which they contribute, including pre-mRNA splicing and processing, and mRNA localization. Expanded repeat RNA and bound RBPs can also aggregate into RNA foci, causing toxicity. Expanded repeat RNA can stall translational complexes, leading to repeat-associated non-AUG (RAN) translation, and contribute to the production of polymeric proteins. Polymeric proteins are aggregate prone. Longer polymeric stretches in native proteins may also cause dysfunction by preventing proper protein folding or causing the folded protein to mis-localize within the cell.

In the previous section, we summarized how STRs in DNA, RNA, and when translated into proteins can affect gene expression and protein function. In the following section, we will draw parallels from these native functions of STRs to pathogenic mechanisms in REDs (Figure 2). These parallels demonstrate how STR toxicity can be viewed as aberrancies of native processes, rather than emergent dysfunctions. For this analysis, we will largely use the Fragile X locus discussed earlier as a well-characterized case study, although many of these principles also apply to other REDs and a few specific examples are included here (reviewed in broader detail in Malik et al., 2021a; Glineburg et al., 2018; Paulson, 2018; Rodriguez and Todd, 2019).

#### Epigenetic and transcriptional dysfunction of STRs in DNA

The functional consequences of STRs on genome organization and transcription are evident when dysfunction is observed in REDs (Dion and Wilson, 2009; López-Martínez et al., 2020; Yin et al., 2020; Usdin, 2008). Repeat expansions can alter local genome architecture and expression of neighboring genes. A prime example is observed at a CTG repeat in the 3’UTR of the *DMPK* gene associated with myotonic dystrophy type 1 (DM1), expansion of which alters local chromatin structure and suppresses transcription of neighboring gene, Six5 (Winchester et al., 1999; Brouwer et al., 2013; López Castel et al., 2011). Repeat expansions also cause global alterations in chromatin structure. CGG repeat expansions in FXS patients cause severe disruptions in chromatin boundaries (Figure 2 (i); Sun et al., 2018). These disruptions may explain delayed DNA replication (Subramanian et al., 1996), activation of DNA replication stress pathways (Chakraborty et al., 2020) and altered local DNA replication patterns (Gerhardt et al., 2014) observed at CGG repeat expansions and the Fragile X locus in particular.

As genomic repeats influence native DNA methylation, some STRs are aberrantly methylated only upon expansion (Figure 2(ii); Otten and Tapscott, 1995; Steinbach et al., 1998; Herman et al., 2006; Greene et al., 2007; Belzil et al., 2013; Xi et al., 2013). When the CGG repeat in the 5’ UTR of *FMR1* expands beyond 200 repeats, it is susceptible to DNA methylation of both the CpG elements within the repeat and at a CpG element within the *FMR1* promoter (Oberlé et al., 1991; Sutcliffe et al., 1992; Pieretti et al., 1991; McConkie-Rosell et al., 1993; Hansen et al., 1992; Coffee et al., 2002; Colak et al., 2014; Willemsen et al., 2002). This hypermethylation is associated with *FMR1* gene silencing, with a resulting absence of *FMR1* mRNA and FMRP, a critical RBP involved in synaptic plasticity and neuronal function (Oberlé et al., 1991; Hagerman et al., 2017; Quartier et al., 2017; Myrick et al., 2014). How exactly repeat expansion triggers methylation and the relationship between expansion, methylation, and epigenetic silencing is not fully understood, but the locus remains transcriptionally active and unmethylated in human embryonic stem cells even in the presence of very large repeat expansions, with silencing occurring during differentiation. Some studies suggest that *FMR1* silencing requires co-transcriptional binding of CGG repeat mRNA directly to the *FMR1* promoter region as an RNA-DNA heteroduplex (Colak et al., 2014; Groh et al., 2014).

STR expansions can enhance or inhibit mRNA production from nearby genes. At *FMR1*, premutation range CGG repeats which cause FXTAS or FXPOI (and which are unmethylated) result in elevated transcription of *FMR1* mRNA (Tassone et al., 2000a; Tassone et al., 2000b; Entezam et al., 2007; Brouwer et al., 2008; Kenneson et al., 2001). This may result from use of additional upstream transcription start sites (Beilina et al., 2004; Tassone et al., 2011), or be associated with enrichment of acetylated histones or other chromatin activating factors at the premutation allele (Todd et al., 2010). It’s possible that both hypo-expression and hyperexpression of *FMR1* stems from the complex structures formed by these CGG repeats as DNA (Usdin and Woodford, 1995; Fry and Loeb, 1994; Kettani et al., 1995; Patel et al., 2000). As seen in native STRs, different structures formed by expanded STRs could facilitate or block binding of histone-modifying methylases, demethylases, acetylases, deacetylases, and even entire nucleosomes to affect downstream gene expression (Figure 2 (iii-v)) (Wang et al., 1996; Usdin and Kumari, 2015).

#### Repeat expansions cause defects in pre-mRNA processing and mRNA localization

The native roles of STRs in RNA in regulating splicing mirror splicing dysfunction observed in numerous REDs (Figure 2 (vi)). Splicing of the *HTT* huntingtin gene, which contains a CAG repeat, is altered at expanded repeats associated with Huntington’s Disease, resulting in the production of a transcript containing only exon 1 and the production of an exon 1 HTT protein (Gipson et al., 2013; Sathasivam et al., 2013; Neueder et al., 2017; Neueder et al., 2018; Franich et al., 2019). The exon 1 HTT protein is found in patient tissues and is toxic in model systems (Gipson et al., 2013; Sathasivam et al., 2013; Neueder et al., 2017; Neueder et al., 2018; Franich et al., 2019). Incomplete splicing of *HTT* with the CAG repeat expansion increased with overexpression and decreased with knockdown of splicing factor SRSF6. SRSF6 is predicted to bind to the 5’ end of *HTT* transcripts via the CAG repeat, suggesting that SRSF6-CAG repeat interactions interfere with spliceosome formation at the nearby splice site (Neueder et al., 2018).

Global splicing defects in REDs result from sequestration of critical splicing factors that bind to STR-containing RNA (López-Martínez et al., 2020; Mykowska et al., 2011; Botta et al., 2008). STRs can be binding sites for RBPs, regulating their availability throughout the cell. RNAs with longer STRs bind more RBPs, which can cause a global cellular depletion of these factors (Figure 2 (vii)) (Malik et al., 2021a; Glineburg et al., 2018; Rodriguez and Todd, 2019). In myotonic dystrophy type 1 (DM1), the expanded CTG repeat in the 3’ UTR of the *DMPK* gene (López-Martínez et al., 2020; Korade-Mirnics et al., 1998; Udd and Krahe, 2012) binds to muscleblind-like splicing regulator 1 protein (MBNL1) among other RBPs (Paul et al., 2011; Jiang et al., 2004; Fardaei et al., 2001; Mankodi et al., 2001; Miller et al., 2000), resulting in depletion of critical splicing factors (Botta et al., 2008; Jiang et al., 2004; Pascual et al., 2006; Jog et al., 2012) and global splicing defects (López-Martínez et al., 2020; Paul et al., 2011; Jiang et al., 2004; Du et al., 2010; Philips et al., 1998). RBP depletion by the CTG repeat in DM1 can also impact other aspects of pre-mRNA processing, including polyadenylation (Thomas et al., 2017; Goodwin et al., 2015).

In FXTAS, premutation expansion mRNA sequesters and depletes multiple RBPs that bind to the CGG repeat RNA directly (i.e. DGCR8 Sellier et al., 2013), Purα (Jin et al., 2007), hnRNP A2/B1 (Jin et al., 2007; Iwahashi et al., 2006; Muslimov et al., 2011; Sofola et al., 2007) or indirectly via binding to CGG-bound proteins (i.e. Drosha Sellier et al., 2013 and Sam68 Sellier et al., 2010). These RBPs are involved in a variety of functions that are affected by their sequestration, including miRNA processing (DGCR8, Drosha), mRNA transport (hnRNP A2/B1, Purα) (Figure 2 (viii)), and in splicing (hnRNP A2/B1, Sam68). Splicing defects have been observed in CGG premutation expansion models (Sellier et al., 2010; He et al., 2014), but compensatory overexpression of CGG repeat-sequestered RBPs (Jin et al., 2007; Sofola et al., 2007; He et al., 2014; Qurashi et al., 2011) or blocking RBP binding to CGG repeats (Disney et al., 2012; Verma et al., 2019; Verma et al., 2020; Verma et al., 2022) can improve these disease-associated defects. This pathogenic sequestration of RBPs by expanded repeats mirrors the native role for STRs in RNA as RBP reserves, mediating fine-tuned dosing of RBP availability with repeat length.

In addition to the depletion of RBPs and consequent defects in RNA splicing and localization and miRNA processing, expanded STRs in RNAs may also cause toxicity by self-association (gelation) (Figure 2(ix); Glineburg et al., 2018; Sellier et al., 2010; He et al., 2014; Jain and Vale, 2017; Ciesiolka et al., 2017; Fay et al., 2017; Tassone et al., 2004). Yet, these processes also occur on RNAs with shorter STRs that are below the pathological threshold for disease, suggesting such that such phase separation properties of specific RNA motifs and their associated RBPs may exist on a spectrum from physiologic to pathologic.

Expanded STRs in RNA can mis-localize or be retained in the nucleus instead of transported to its functional location in the cell (Davis et al., 1997; Mastroyiannopoulos et al., 2010; Sun et al., 2015). This may be mediated by splicing defects (Sun et al., 2015), via export-inhibiting RBP interactions (Smith et al., 2007), or via a larger dysfunction of nucleocytoplasmic transport (Zhang et al., 2015; Zhang et al., 2016; Jovičić et al., 2015; Freibaum et al., 2015; Grima et al., 2017; Gasset-Rosa et al., 2017; Sellier et al., 2017). For example, SRSF proteins bind to CGG and G4C2 repeats and appear critical to their cytoplasmic transport out of the nucleus (Malik et al., 2021b; Hautbergue et al., 2017). In this context, lowered expression of SRSF proteins or inhibition of the SRSF protein kinase SRPK1, which regulates SRSF nuclear entry, suppress CGG repeat exit to the cytoplasm and reduce toxicity in *Drosophila* and neuronal model systems (Malik et al., 2021b). Together, these studies show that expanded STRs in RNA can induce toxicity via RBP depletion or by direct RNA dysfunction.

#### Aberrant translation of expanded STRs

Scanning translational complexes are more likely to stall at stable secondary structures formed by expanded STRs (Figure 2 (x)), resulting in aberrant translation initiation upstream of or within the repeat in a process known as repeat-associated non-AUG (RAN) translation (Figure 2 (xi)). RAN translation produces toxic peptides that contribute to expanded STR toxicity and neurodegeneration in numerous REDs (Glineburg et al., 2018; Ciesiolka et al., 2017; Cleary and Ranum, 2017; Kearse and Todd, 2014; Kearse et al., 2016; Todd et al., 2013; Wojciechowska et al., 2014; Mori et al., 2013a; Ash et al., 2013; Mori et al., 2013b; Bañez-Coronel et al., 2015; Zu et al., 2011; Zu et al., 2017; Soragni et al., 2018; Ishiguro et al., 2017).

The mechanisms underlying RAN translation likely vary across different STRs and different genetic contexts (Malik et al., 2021a; Gao et al., 2017). At the CGG repeat of *FMR1*, RAN translation occurs in all three reading frames to produce polyarginine (FMRpolyR) (+0-frame relative to the AUG of *FMR1*), polyglycine (FMRpolyG) (+1-frame), and polyalanine (FMRpolyA) (+2-frame) peptides at different efficiencies (Kearse et al., 2016; Todd et al., 2013). STR-induced stalling of translation machinery is also responsible for a reduction in downstream production of the main protein produced by *FMR1* translation, FMRP, in CGG premutation carriers (Tassone et al., 2000b; Kenneson et al., 2001).

#### Repetitive proteins have pathogenic consequences

Repeat-containing peptides, produced via canonical translation of STRs in coding regions or via RAN translation, contribute to toxicity in REDs. At CGG repeats, both FMRpolyG and FMRpolyA are present within intranuclear neuronal inclusions in patient tissues (Sellier et al., 2017; Todd et al., 2013; Buijsen et al., 2014; Krans et al., 2019; Ma et al., 2019), and are toxic in model systems (Sellier et al., 2017; Todd et al., 2013; Derbis et al., 2018; Gohel et al., 2019; Hoem et al., 2019). FMRpolyG, the most abundant CGG RAN product, is necessary for CGG repeat toxicity and inclusion formation (Sellier et al., 2017; Todd et al., 2013; Oh et al., 2015) in overexpression models. Numerous RAN or homopolymeric peptides generated in other REDs are essential for their toxicity and formation of proteinaceous inclusions (Figure 2 (xii); Yamamoto et al., 2000; Schilling et al., 1999; Ordway et al., 1997; Bäuerlein et al., 2017; Paulson et al., 1997; Mizielinska et al., 2014; May et al., 2014; Zu et al., 2013). Overall, dysfunctional aggregation of repeat derived protein products mirrors the native function of STRs in proteins as facilitators of protein-protein interactions.

Translation through large STRs that form stable secondary structures likely induces ribosome stalls and elongation errors. A growing body of work shows that disease-associated STRs undergo stall-induced translational frameshifting to produce novel chimeric polypeptides (Gaspar et al., 2000; Toulouse et al., 2005; Davies and Rubinsztein, 2006; Tabet et al., 2018; McEachin et al., 2020; Wright et al., 2022), and several of these studies have shown that these frameshift products have distinct contributions to neuronal dysfunction in disease (Tabet et al., 2018; McEachin et al., 2020; Wright et al., 2022). While there is evidence that polymeric peptides contribute to toxicity observed in REDs via aggregation, the mechanistic details of homo- and di-polymeric peptide toxicity and chimeric polypeptide toxicity remain under investigation.

#### Antisense transcripts contribute to REDs via multiple mechanisms

Antisense transcription from the *FMR1* locus generates multiple long-noncoding as*FMR1* mRNAs, with some including the repeat (Ladd et al., 2007; Khalil et al., 2008; Elizur et al., 2016; Pastori et al., 2014). One antisense transcript, *FMR4*, is thought to play a critical role in regulating the cell cycle and apoptosis (Khalil et al., 2008). Another antisense transcript, *FMR6*, is upregulated in premutation women, with increased repeat length correlating to elevated RNA levels and reduced number of oocytes, suggesting a relationship between antisense transcript expression and toxicity (Elizur et al., 2016). *FMR1* antisense transcription in general is upregulated in Fragile X premutation disorders and lost in FXS, like the sense *FMR1* mRNA (Ladd et al., 2007). Moreover, *asFMR1* mRNAs containing the CCG repeats can undergo RAN translation, producing additional homopolymeric proteins with toxic potential (Kearse et al., 2016). STR-containing antisense transcripts likely contribute to toxicity observed in many REDs, but this is best characterized in *C9ALS/FTD* and *SCA8*, where antisense transcripts are found in toxic RNA foci and contribute to RAN peptide production (Mori et al., 2013a; Zu et al., 2011; Zu et al., 2013; Moseley et al., 2006; Gendron et al., 2013).

### Mechanisms of STR toxicity reveal novel native functions of STRs

Studies over the past three decades have delineated numerous mechanisms by which repeat expansions trigger cellular toxicity. Yet, there are striking parallels between the pathologic drivers of dysfunction elicited by repeat expansions and the native functions of STRs in regulating gene expression. In this section, we provide examples of how mechanisms initially identified as causing STR toxicity directly inform our understanding of native functions of STRs more broadly. We also discuss how emergent pathogenic properties associated with repeat expansions might inform additional native functions of repeats that are not yet well understood.

#### RAN translation occurs at native repeat lengths and have native functions

While CGG repeats in the *FMR1* gene were primarily studied for their disease association, the CGG repeat is present in all humans at nonpathogenic lengths (<55 repeats) and conserved across mammals (Eichler et al., 1995; Sellier et al., 2017). Some studies suggest phenotypes associated with low CGG repeat numbers at this allele in humans, including memory difficulties and language dysfluency (Klusek et al., 2018; Mailick et al., 2014). Our group observed that CGG RAN translation, originally thought to be an aberrant toxic event, occurs in reporters with native repeat lengths (25 repeats) (Kearse et al., 2016), suggesting CGG repeats and/or translation of those repeats may have a native function in addition to the pathogenic one. CGG RAN translation at native and expanded STRs acts as an overlapping upstream open reading frame (uORF), inhibiting translation of the downstream main ORF (mORF) and thereby reducing FMRP synthesis (Rodriguez et al., 2020). Furthermore, this RAN uORF-like regulation of FMRP synthesis was critical for facilitating translational changes associated with stimulation of metabotropic glutamate receptors (mGluRs) in neurons (Rodriguez et al., 2020).

Upstream open-reading frames (uORFs) are well-characterized regulatory elements in eukaryotes that influence expression of protein produced from the main open reading frame (mORF) on the same transcript, and are typically inhibitory to downstream mORF translation (Hinnebusch et al., 2016). In this way, uORFs resulting from RAN translation of STRs may play a global role in regulating mRNA translation, presenting another mechanism by which STRs influence gene expression.

#### STRs facilitate protein function and localization

Expanded STRs in coding regions can fundamentally change the functions of the proteins within which they reside. In spinocerebellar ataxia type 1 (SCA1), a CAG repeat expansion in the ataxin 1 (*ATXN1*) gene changes the localization of ATXN1 protein (Irwin et al., 2005). ATXN1 normally shuttles between the nucleus and the cytoplasm, but an expanded polyQ region shifts ATXN1 localization to the nucleus (Figure 2(xiii); Irwin et al., 2005). Aberrant nuclear localization of ATXN1 underlies dysfunction in SCA1 (Lam et al., 2006; Lai et al., 2011; Klement et al., 1998; Emamian et al., 2003; Duvick et al., 2010), as modifications that favor nuclear localization are sufficient to elicit disease relevant phenotypes in the absence of the repeat expansion in mouse models.

PolyQ-associated nuclear translocation is also central to pathology in spinal and bulbar muscular atrophy (SBMA), where ligand binding and translocation to the nucleus of the expanded PolyQ-containing androgen receptor is required to elicit disease-associated transcriptional defects and cytotoxicity (Katsuno et al., 2006; Katsuno et al., 2002; Montie et al., 2009; Palazzolo et al., 2007). However, within the normal range of polyQ lengths observed in humans, Androgen receptor CAG repeat size inversely correlates with the receptor’s transactivational activity and linearly correlates with infertility and decreased sperm function (Choong and Wilson, 1998; Osadchuk et al., 2022; Pan et al., 2016). These findings suggest that the CAG repeats play a normal role in testosterone activated gene cascades that become aberrant at larger repeat sizes.

#### STRs facilitate mRNA transport to dendrites

An investigation into dendritic mRNA localization identified a localization pathway dependent on the interaction of a CGG repeat-interacting RBP, hnRNP A2, with a GA dendritic targeting element of an RNA (Muslimov et al., 2011). This GA targeting motif was competed for by CGG repeat-containing RNAs, including *FMR1* mRNA. In addition to a native function of CGG repeats as a dendritic localization factor, this study revealed that elevated levels of CGG repeat mRNA caused by the CGG premutation expansion sequester hnRNP A2, resulting in global dysfunction in the transport of hnRNP A2-target mRNAs (Muslimov et al., 2011). Another study seeking to reveal transcriptome-wide impacts of C(C)UG repeat-mediated MBNL depletion on splicing in myotonic dystrophy (DM) also uncovered a global role for MBNL in mRNA localization (Wang et al., 2012).

#### PolyQ containing proteins regulate autophagy

Numerous REDs are caused by CAG repeats, including the huntingtin gene in Huntington’s disease (HD) and Ataxin 3 in spinocerebellar ataxia type 3 (SCA3), with toxicity largely attributed to the aggregation of long polyQ containing proteins. Autophagy induction results in clearance of these aggregates, attenuating their toxicity (Rubinsztein, 2006; Ravikumar et al., 2004; Menzies et al., 2010). PolyQ tracts in ataxin 3, a deubiquitinase associated with spinocerebellar ataxia type 3 (SCA3), interact with beclin 1, a key initiator of autophagy (Ashkenazi et al., 2017). Ataxin 3 then deuniquitinates beclin 1, protecting it from degradation and permitting autophagy initiation. Ataxin 3 activity and interaction with beclin 1 is competitively inhibited by other polyQ tract-containing proteins in a length-dependent manner (Ashkenazi et al., 2017). As such, polyQ tracts may actively engage protein quality control pathways basally but then these interactions become aberrant after STR expansion, in this case inhibiting autophagy and clearance of toxic proteins. Together, these studies suggest that the pathology of disease-associated STR expansions reveal native functions of STRs, just as an improved understanding of the native functions of STRs can inform on dysfunctions in disease.

#### Tetranucleotide, pentanucleotide, and biallelic repeat expansion disorders

Tetranucleotide and pentanucleotide STRs are rare within coding sequences, presumably because changes in their repeat number would trigger translational frameshifts. However, they are relatively common within introns, where their expansion causes several neurological disorders that likely act through pathogenic mechanisms that are similar to those exhibited by non-coding trinucleotide STRs. For example, Myotonic dystrophy type 2 (DM2) results from a dominantly inherited intronic CCTG STR expansion in *ZNF9* (Liquori et al., 2001). CCTG STRs form RNA secondary structures that are like those generated by CTG STRs, and like the 3’ UTR CTG repeat in DM1, the DM2 repeat binds to and sequesters the RBP muscleblind (Botta et al., 2008; Paul et al., 2011; Fardaei et al., 2001; Mankodi et al., 2001; Miller et al., 2000; Du et al., 2010; Philips et al., 1998). This shared mechanism explains the significant overlap in their clinical phenotypes. Perhaps more interesting, however, is how subtle differences in how these repeats underlie the phenotypic differences in these conditions. In particular, CCTG expansions in DM2 do not trigger genetic anticipation or congenital forms of disease as occurs in DM1 despite the presence of very large CCTG expansions in DM2. These phenotypic differences are thought to occur for two reasons. First, these repeats exhibit differences in how they interact with other RBPs, such as rbFOX, that modulate the effects of muscleblind sequestration (Sellier et al., 2018). Second, differences in the genic positioning (intron versus 3’ UTR) and temporal expression of the two STRs alters their relative abilities to disrupt early developmental processes (Thomas et al., 2017; Cerro-Herreros et al., 2017).

An intriguing feature observed in multiple pentanucleotide repeat expansion disorders, including complex TTTTA a d TTTCA repeats that cause benign adult familial myoclonic epilepsy (BAFME) in multiple genes (Ishiura et al., 2018), ATTTC repeats in Spinocerebellar ataxia (SCA) type 31 (Sato et al., 2009), and AAGGG repeats that cause cerebellar ataxia, neuropathy, vestibular areflexia syndrome (CANVAS) (Nakamura et al., 2020; Cortese et al., 2019; Tsuchiya et al., 2020), is that the pathogenic alleles represent non-reference STRs. That is, the repeat element is not only expanded in size, but it has a different sequence than the normal allele. For example, in CANVAS, an AAAAG pentanucleotide STR normally resides within the first intron of *RFC1*. However, the pathological repeat is a qualitatively different and expanded AAGGG repeat. Moreover, CANVAS can also occur with a third pentanucleotide repeat, ACAGG, at this same genomic location. In all these cases, these pentanucleotide repeats occur within the polyA region of an Alu transposable element. Active Alu transposition requires pure polyA elements at their 3’ ends (Deininger, 2011). As such, there is strong evolutionary selection pressure favoring mutation of these regions to pure polyA sequences. This suggests that both the reference and non-reference STRs occurred initially through a protective process that disrupted the polyA element and prevented continued transposase activity. However, stochastic differences in these interrupting mutations created some STRs that were more prone to expansion, resulting in pathogenic alleles that either create toxic STR RNAs or that interfere with local gene expression.

### Conclusions and open questions

#### Native functions of STRs from an evolutionary perspective

**Figure 3.**
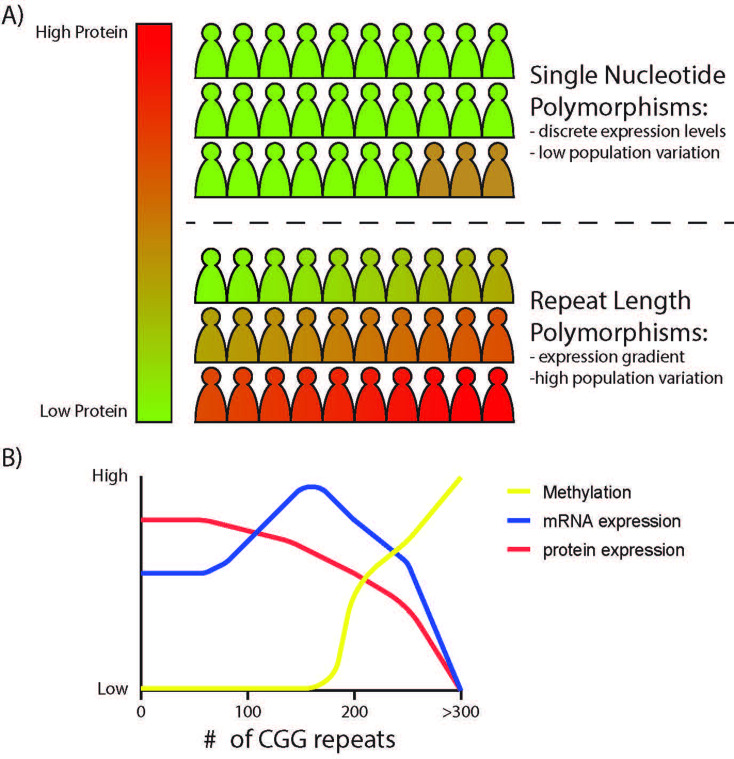
The effects of STR variation on local gene expressivity. (**A**) Bi-allelic variation in a gene through single nucleotide polymorphisms often result in small and discrete differences in gene expression, offering limited phenotypic differences across a population with slow evolutionary timescales. In contrast, STRs in promoters and 5’UTRs can influence protein expression over a broader dynamic range, with an inverse correlation between repeat length and protein output within transcribed regions and with differential effects on transcription dependent on the repeat and local epigenetic context. Unstable repeats change rapidly from generation to generation (and even within an individual through somatic variation), creating a mechanism by which mRNA or protein expression can vary broadly and subtly across a population, offering greater genetic and phenotypic diversity and a greater propensity for disease-causing aberrancies at the extremes. (**B**) Predicted effects of CGG repeat length on FMR1 gene expression. CGG repeat length influences FMR1 promoter epigenetic state (more open chromatin with initial expansion, then DNA methylation and closed chromatin at >200 CGG repeats), FMR1 mRNA expression, and FMRP protein expression across the polymorphic range.

Evolutionary pressures on STR copy number predict that repeat expansions will be either tolerated or selected for until an upper, deleterious limit is reached. If STRs were intrinsically deleterious, then there would be selective pressure towards repeat contractions, leading to global reductions in STR size or their selective elimination. However, several recent studies suggest that there is selective STR expansion across phylogeny, especially in primates. This is particularly true in 5’ UTRs and coding regions, where constraints on repeat expansion and contraction are greatest (Gymrek et al., 2017). At the same time, many intergenic STRs show correlations between their size and the expression of neighboring genes. These eSTRs contribute meaningfully to population variance in gene expression profiles and disease associated Quantitative trait loci (eQTLs) in human populations (Fotsing et al., 2019; Gymrek et al., 2017). In general, these eSTRs are largely unconstrained unless neighboring or embedded within a gene already under strong constraint. This mutation-selection balance suggests some inherent native functions of STRs within transcript and protein space while also implying that intrinsic STR instability may allow for more rapid variation and acquisition of traits through local perturbations in gene expression than could be accomplished through single nucleotide mutations (Figure 3A). The highly variable methylation status, mRNA and protein expression patterns elicited by differences in *FMR1* CGG repeat lengths typify the potential for repeat variation within populations to influence gene expression (Figure 3B). As even subtle changes in repeat size can tune gene expression and protein function and have downstream impacts on simple and complex phenotypes, they may be an important component of the genetic differences between humans and other species, and among humans themselves.

#### Revisiting our understanding of and approach to Repeat Expansion Disorders

Historically, repetitive elements within human genomes have been viewed as mostly unregulated ‘junk DNA’ that is not under selective evolutionary pressure. As such expansions of these repetitive elements are unfortunate accidents which become apparent and important only when they elicit highly penetrant and syndromic human diseases. Consistent with this line of reasoning, the field of REDs has largely focused on emergent toxic mechanisms as drivers of disease only in the setting of large STR expansions rather than considering their pathology as alterations in the native functions played by these repeats in their normal genomic contexts. Here, we propose re-framing the discussion around repetitive elements in general- and STRs in particular- within human genomes. For each STR, we suggest first considering whether the STRs associated with a human disease have any native functions at their ‘normal’ size. If a native function exists, then expansion of these STRs can be viewed primarily as an aberrancy of that native function with coincident predictable impacts on gene expression dysregulation above certain repeat lengths. This reframing aligns with the approach typically taken in studying gain-of-function and loss-of-function mutations in disease associated single amino acid mutations and better ties the native functions of STRs to their pathology. It also suggests that shared regulatory rules will likely apply across REDs.

This approach to thinking about REDs leads to specific predictions. First, we predict that more REDs will be discovered in the future. For example, multiple recently described REDs are linked to CGG repeats, including neuronal intranuclear inclusion disease (NIID) (Ishiura et al., 2019; Sone et al., 2019), oculopharyngodistal myopathy (OPDM) and leukodystrophy (OPML) (Ishiura et al., 2019; Deng et al., 2020; Ogasawara et al., 2020; Tian et al., 2019), adult onset leukoencephalopathy (Okubo et al., 2019), and autism/intellectual disability (Annear et al., 2022). Most of these new CGG repeatopathies reside within the 5’ UTRs, like the CGG repeat in *FMR1*, and there is already evidence of convergent disease mechanisms triggered by these new repeats with those already established in Fragile X disorders. In one particularly notable example, a CGG repeat expansion in *NOTCH2NLC* leads to the creation of an AUG-initiated upstream open reading frame in the 5’ UTR that is generates a polyglycine-containing protein akin to FMRpolyG in FMR1 (Liu et al., 2022; Boivin et al., 2021). This polyglycine protein is found within inclusions in patients with NIID and its generation is required to trigger inclusion formation and behavioral phenotypes in a mouse model of *NOTCH2NLC* associated NIID. As such, we know that this motif in this location within neuronally expressed genes can elicit dysfunction through predictable mechanisms. This means that we should expect other CGG repeat expansions to emerge that mirror the pathologic processes established for the FMR1 locus and now extended to a large set of loci. Similarly, given evidence that the CGG repeat in *FMR1* 5’UTRs can serve as a functional element that regulates transcription, mRNA localization and translation, we predict that native CGG repeat elements in these disease-associated alleles may have normal functions akin to those observed for *FMR1*, and as such represent a functional motif shared among many genes.

However, these new REDs may not all fit the typical model observed to date, where highly penetrant STR expansions lead to syndromic disorders. Instead, smaller changes in repeat size at multiple loci, impacting expression of the genes in which they reside or neighboring genes, will serve as risk alleles for common conditions. This risk-allele model is already apparent, as intermediate CAG repeat expansions in *ATXN1*, *ATXN2*, and *HTT* are associated with sporadic ALS and some other common neurodegenerative disorders (Elden et al., 2010; Rosas et al., 2020). Indeed, a fair proportion of the unexplained signal within Genome Wide association Studies (GWAS) can be explained by variations within neighboring STRs (Gymrek et al., 2016; Gymrek, 2017; Hannan, 2018). To date, numerous STR variants have been linked to ASD (Mitra et al., 2021; Trost et al., 2020) and Schizophrenia (Mojarad et al., 2022). As PCR-free and long-read whole genome data becomes more abundant and available (reviewed in Mitsuhashi and Matsumoto, 2020), it will become increasingly easy to detect these dynamic repeat size/disease relationships, creating a whole new class of STR-associated conditions that will likely expand outside of neurological conditions.

Second, we predict that long-read whole genome sequencing datasets will improve our understanding of the native roles of STRs in humans, and reveal a ubiquitous impact of repeat length variation on gene expression. Once we create accurate maps of STR variation across the genome and link this variation to neighboring gene loci expression, we will be able to better discern the mechanisms by which STRs influence gene expression across cell types. We predict that many genes whose expression is affected by neighboring repeat length variation will play critical functions in the nervous system. Most known REDs present with neurological symptoms. If REDs reflect the native functions of STRs, then the overrepresentation of neurological dysfunctions linked to STR expansions suggests that STRs may play roles relevant to neuronal health and function. It is also possible that neurons, as terminally differentiated cells, may be more prone to somatic instability, leading to repeat expansion and the emergence of associated dysfunction with age.

Finally, we predict that the native functions of STRs will inform our understanding of how STR expansions cause disease and vice versa. A deeper understanding of the native functions of both disease-associated STRs and STRs in general will reveal the pathways altered in REDs, and these pathways may be areas for therapeutic intervention that can be applicable across all REDs. By studying the mechanisms by which STRs elicit disease, we can also surmise key elements of how they might function normally within nervous systems (see examples in previous section, “Mechanisms of STR toxicity reveal novel native functions of STRs”). Ultimately, research into native functions of STRs will reveal both mechanisms by which they regulate neuronal function and therapeutic targets by which their toxicity in REDs can be mitigated.

## References

[bib1] Abruzzi KC, Lacadie S, Rosbash M (2004). Biochemical analysis of TREX complex recruitment to intronless and intron-containing yeast genes. The EMBO Journal.

[bib2] Allingham-Hawkins DJ, Babul-Hirji R, Chitayat D, Holden JJ, Yang KT, Lee C, Hudson R, Gorwill H, Nolin SL, Glicksman A, Jenkins EC, Brown WT, Howard-Peebles PN, Becchi C, Cummings E, Fallon L, Seitz S, Black SH, Vianna-Morgante AM, Costa SS, Otto PA, Mingroni-Netto RC, Murray A, Webb J, Vieri F (1999). Fragile X premutation is a significant risk factor for premature ovarian failure: the International collaborative POF in fragile X study -- preliminary data. American Journal of Medical Genetics.

[bib3] Andken BB, Lim I, Benson G, Vincent JJ, Ferenc MT, Heinrich B, Jarzylo LA, Man HY, Deshler JO (2007). 3’-Utr SIRF: a database for identifying clusters of whort interspersed repeats in 3’ untranslated regions. BMC Bioinformatics.

[bib4] Andrade MA, Perez-Iratxeta C, Ponting CP (2001). Protein repeats: structures, functions, and evolution. Journal of Structural Biology.

[bib5] Annear DJ, Vandeweyer G, Sanchis-Juan A, Raymond FL, Kooy RF (2022). Non-Mendelian inheritance patterns and extreme deviation rates of CGG repeats in autism. Genome Research.

[bib6] Ash PEA, Bieniek KF, Gendron TF, Caulfield T, Lin WL, Dejesus-Hernandez M, van Blitterswijk MM, Jansen-West K, Paul JW, Rademakers R, Boylan KB, Dickson DW, Petrucelli L (2013). Unconventional translation of c9orf72 GGGGCC expansion generates insoluble polypeptides specific to c9ftd/ALS. Neuron.

[bib7] Ashkenazi A, Bento CF, Ricketts T, Vicinanza M, Siddiqi F, Pavel M, Squitieri F, Hardenberg MC, Imarisio S, Menzies FM, Rubinsztein DC (2017). Polyglutamine tracts regulate Beclin 1-dependent autophagy. Nature.

[bib8] Aslanidis C, Jansen G, Amemiya C, Shutler G, Mahadevan M, Tsilfidis C, Chen C, Alleman J, Wormskamp NGM, Vooijs M (1992). Cloning of the essential myotonic dystrophy region and mapping of the putative defect. Nature.

[bib9] Bañez-Coronel M, Ayhan F, Tarabochia AD, Zu T, Perez BA, Tusi SK, Pletnikova O, Borchelt DR, Ross CA, Margolis RL, Yachnis AT, Troncoso JC, Ranum LPW (2015). RAN translation in huntington disease. Neuron.

[bib10] Bäuerlein FJB, Saha I, Mishra A, Kalemanov M, Martínez-Sánchez A, Klein R, Dudanova I, Hipp MS, Hartl FU, Baumeister W, Fernández-Busnadiego R (2017). In situ architecture and cellular interactions of polyQ inclusions. Cell.

[bib11] Beilina A, Tassone F, Schwartz PH, Sahota P, Hagerman PJ (2004). Redistribution of transcription start sites within the FMR1 promoter region with expansion of the downstream CGG-repeat element. Human Molecular Genetics.

[bib12] Belotserkovskii BP, Liu R, Tornaletti S, Krasilnikova MM, Mirkin SM, Hanawalt PC (2010). Mechanisms and implications of transcription blockage by guanine-rich DNA sequences. PNAS.

[bib13] Belzil VV, Bauer PO, Prudencio M, Gendron TF, Stetler CT, Yan IK, Pregent L, Daughrity L, Baker MC, Rademakers R, Boylan K, Patel TC, Dickson DW, Petrucelli L (2013). Reduced C9orf72 gene expression in c9FTD/ALS is caused by histone trimethylation, an epigenetic event detectable in blood. Acta Neuropathologica.

[bib14] Bentele K, Saffert P, Rauscher R, Ignatova Z, Blüthgen N (2013). Efficient translation initiation dictates codon usage at gene start. Molecular Systems Biology.

[bib15] Betley JN, Frith MC, Graber JH, Choo S, Deshler JO (2002). A ubiquitous and conserved signal for RNA localization in chordates. Current Biology.

[bib16] Black DL (2003). Mechanisms of alternative pre-messenger RNA splicing. Annual Review of Biochemistry.

[bib17] Blackledge NP, Thomson JP, Skene PJ (2013). Cpg island chromatin is shaped by recruitment of ZF-cxxc proteins. Cold Spring Harbor Perspectives in Biology.

[bib18] Boivin M, Deng J, Pfister V, Grandgirard E, Oulad-Abdelghani M, Morlet B, Ruffenach F, Negroni L, Koebel P, Jacob H, Riet F, Dijkstra AA, McFadden K, Clayton WA, Hong D, Miyahara H, Iwasaki Y, Sone J, Wang Z, Charlet-Berguerand N (2021). Translation of GGC repeat expansions into a toxic polyglycine protein in NIID defines a novel class of human genetic disorders: the polyg diseases. Neuron.

[bib19] Bolton KA, Ross JP, Grice DM, Bowden NA, Holliday EG, Avery-Kiejda KA, Scott RJ (2013). STaRRRT: a table of short tandem repeats in regulatory regions of the human genome. BMC Genomics.

[bib20] Botta A, Rinaldi F, Catalli C, Vergani L, Bonifazi E, Romeo V, Loro E, Viola A, Angelini C, Novelli G (2008). The CTG repeat expansion size correlates with the splicing defects observed in muscles from myotonic dystrophy type 1 patients. Journal of Medical Genetics.

[bib21] Bourque G (2009). Transposable elements in gene regulation and in the evolution of vertebrate genomes. Current Opinion in Genetics & Development.

[bib22] Britten RJ, Kohne DE (1968). Repeated sequences in DNA: hundreds of thousands of copies of DNA sequences have been incorporated into the genomes of higher organisms. Science.

[bib23] Brook JD, McCurrach ME, Harley HG, Buckler AJ, Church D, Aburatani H, Hunter K, Stanton VP, Thirion JP, Hudson T (1992). Molecular basis of myotonic dystrophy: expansion of a trinucleotide (CTG) repeat at the 3’ end of a transcript encoding a protein kinase family member. Cell.

[bib24] Brouwer JR, Huizer K, Severijnen LA, Hukema RK, Berman RF, Oostra BA, Willemsen R (2008). Cgg-Repeat length and neuropathological and molecular correlates in a mouse model for fragile X-associated tremor/ataxia syndrome. Journal of Neurochemistry.

[bib25] Brouwer JR, Huguet A, Nicole A, Munnich A, Gourdon G (2013). Transcriptionally repressive chromatin remodelling and CpG methylation in the presence of expanded CTG-repeats at the DM1 locus. Journal of Nucleic Acids.

[bib26] Brunberg JA, Jacquemont S, Hagerman RJ, Berry-Kravis EM, Grigsby J, Leehey MA, Tassone F, Brown WT, Greco CM, Hagerman PJ (2002). Fragile X premutation carriers: characteristic MR imaging findings of adult male patients with progressive cerebellar and cognitive dysfunction. AJNR. American Journal of Neuroradiology.

[bib27] Buijsen RAM, Sellier C, Severijnen L, Oulad-Abdelghani M, Verhagen RFM, Berman RF, Charlet-Berguerand N, Willemsen R, Hukema RK (2014). FMRpolyG-positive inclusions in CNS and non-CNS organs of a fragile X premutation carrier with fragile X-associated tremor/ataxia syndrome. Acta Neuropathologica Communications.

[bib28] Buxton J, Shelbourne P, Davies J, Jones C, Van Tongeren T, Aslanidis C, de Jong P, Jansen G, Anvret M, Riley B (1992). Detection of an unstable fragment of DNA specific to individuals with myotonic dystrophy. Nature.

[bib29] Caron NS, Desmond CR, Xia J, Truant R (2013). Polyglutamine domain flexibility mediates the proximity between flanking sequences in huntingtin. PNAS.

[bib30] Cerro-Herreros E, Chakraborty M, Pérez-Alonso M, Artero R, Llamusí B (2017). Expanded CCUG repeat RNA expression in *Drosophila* heart and muscle trigger myotonic dystrophy type 1-like phenotypes and activate autophagocytosis genes. Scientific Reports.

[bib31] Chakraborty A, Jenjaroenpun P, Li J, El Hilali S, McCulley A, Haarer B, Hoffman EA, Belak A, Thorland A, Hehnly H, Schildkraut CL, Chen CL, Kuznetsov VA, Feng W (2020). Replication stress induces global chromosome breakage in the fragile X genome. Cell Reports.

[bib32] Chávez S, Beilharz T, Rondón AG, Erdjument-Bromage H, Tempst P, Svejstrup JQ, Lithgow T, Aguilera A (2000). A protein complex containing tho2, hpr1, mft1 and a novel protein, thp2, connects transcription elongation with mitotic recombination in *Saccharomyces cerevisiae*. The EMBO Journal.

[bib33] Chávez S, García-Rubio M, Prado F, Aguilera A (2001). Hpr1 is preferentially required for transcription of either long or G+C-rich DNA sequences in *Saccharomyces cerevisiae*. Molecular and Cellular Biology.

[bib34] Choong CS, Wilson EM (1998). Trinucleotide repeats in the human androgen receptor: a molecular basis for disease. Journal of Molecular Endocrinology.

[bib35] Chudley AE, Hagerman RJ (1987). Fragile X syndrome. The Journal of Pediatrics.

[bib36] Ciesiolka A, Jazurek M, Drazkowska K, Krzyzosiak WJ (2017). Structural characteristics of simple RNA repeats associated with disease and their deleterious protein interactions. Frontiers in Cellular Neuroscience.

[bib37] Cleary JD, Ranum LP (2017). New developments in Ran translation: insights from multiple diseases. Current Opinion in Genetics & Development.

[bib38] Clouaire T, Webb S, Skene P, Illingworth R, Kerr A, Andrews R, Lee JH, Skalnik D, Bird A (2012). Cfp1 integrates both CpG content and gene activity for accurate H3K4me3 deposition in embryonic stem cells. Genes & Development.

[bib39] Coffee B, Zhang F, Ceman S, Warren ST, Reines D (2002). Histone modifications depict an aberrantly heterochromatinized FMR1 gene in fragile X syndrome. American Journal of Human Genetics.

[bib40] Colak D, Zaninovic N, Cohen MS, Rosenwaks Z, Yang WY, Gerhardt J, Disney MD, Jaffrey SR (2014). Promoter-Bound trinucleotide repeat mRNA drives epigenetic silencing in fragile X syndrome. Science.

[bib41] Comfort NC (2001). From controlling elements to transposons: Barbara McClintock and the Nobel Prize. Trends in Genetics.

[bib42] Cortese A, Simone R, Sullivan R, Vandrovcova J, Tariq H, Yau WY, Humphrey J, Jaunmuktane Z, Sivakumar P, Polke J, Ilyas M, Tribollet E, Tomaselli PJ, Devigili G, Callegari I, Versino M, Salpietro V, Efthymiou S, Kaski D, Wood NW, Andrade NS, Buglo E, Rebelo A, Rossor AM, Bronstein A, Fratta P, Marques WJ, Züchner S, Reilly MM, Houlden H (2019). Biallelic expansion of an intronic repeat in RFC1 is a common cause of late-onset ataxia. Nature Genetics.

[bib43] Cournac A, Koszul R, Mozziconacci J (2016). The 3D folding of metazoan genomes correlates with the association of similar repetitive elements. Nucleic Acids Research.

[bib44] Davies JE, Rubinsztein DC (2006). Polyalanine and polyserine frameshift products in Huntington’s disease. Journal of Medical Genetics.

[bib45] Davis BM, McCurrach ME, Taneja KL, Singer RH, Housman DE (1997). Expansion of a CUG trinucleotide repeat in the 3’ untranslated region of myotonic dystrophy protein kinase transcripts results in nuclear retention of transcripts. PNAS.

[bib46] Deaton AM, Bird A (2011). Cpg islands and the regulation of transcription. Genes & Development.

[bib47] Debacker K, Kooy RF (2007). Fragile sites and human disease. Human Molecular Genetics.

[bib48] Deininger P (2011). Alu elements: know the SINEs. Genome Biology.

[bib49] de Koning APJ, Gu W, Castoe TA, Batzer MA, Pollock DD (2011). Repetitive elements may comprise over two-thirds of the human genome. PLOS Genetics.

[bib50] Deng J, Yu J, Li P, Luan X, Cao L, Zhao J, Yu M, Zhang W, Lv H, Xie Z, Meng L, Zheng Y, Zhao Y, Gang Q, Wang Q, Liu J, Zhu M, Guo X, Su Y, Liang Y, Liang F, Hayashi T, Maeda MH, Sato T, Ura S, Oya Y, Ogasawara M, Iida A, Nishino I, Zhou C, Yan C, Yuan Y, Hong D, Wang Z (2020). Expansion of GGC repeat in GIPC1 is associated with oculopharyngodistal myopathy. American Journal of Human Genetics.

[bib51] Derbis M, Konieczny P, Walczak A, Sekrecki M, Sobczak K (2018). Quantitative evaluation of toxic polyglycine biosynthesis and aggregation in cell models expressing expanded CGG repeats. Frontiers in Genetics.

[bib52] Ding Y, Shah P, Plotkin JB (2012). Weak 5’-mrna secondary structures in short eukaryotic genes. Genome Biology and Evolution.

[bib53] Dion V, Wilson JH (2009). Instability and chromatin structure of expanded trinucleotide repeats. Trends in Genetics.

[bib54] Disney MD, Liu B, Yang WY, Sellier C, Tran T, Charlet-Berguerand N, Childs-Disney JL (2012). A small molecule that targets R (CGG) (EXP) and improves defects in fragile X-associated tremor ataxia syndrome. ACS Chemical Biology.

[bib55] Du H, Cline MS, Osborne RJ, Tuttle DL, Clark TA, Donohue JP, Hall MP, Shiue L, Swanson MS, Thornton CA, Ares M (2010). Aberrant alternative splicing and extracellular matrix gene expression in mouse models of myotonic dystrophy. Nature Structural & Molecular Biology.

[bib56] Duvick L, Barnes J, Ebner B, Agrawal S, Andresen M, Lim J, Giesler GJ, Zoghbi HY, Orr HT (2010). SCA1-like disease in mice expressing wild-type ataxin-1 with a serine to aspartic acid replacement at residue 776. Neuron.

[bib57] Eichler EE, Kunst CB, Lugenbeel KA, Ryder OA, Davison D, Warren ST, Nelson DL (1995). Evolution of the cryptic FMR1 CGG repeat. Nature Genetics.

[bib58] Elden AC, Kim H-J, Hart MP, Chen-Plotkin AS, Johnson BS, Fang X, Armakola M, Geser F, Greene R, Lu MM, Padmanabhan A, Clay-Falcone D, McCluskey L, Elman L, Juhr D, Gruber PJ, Rüb U, Auburger G, Trojanowski JQ, Lee VM-Y, Van Deerlin VM, Bonini NM, Gitler AD (2010). Ataxin-2 intermediate-length polyglutamine expansions are associated with increased risk for ALS. Nature.

[bib59] Elizur SE, Dratviman-Storobinsky O, Derech-Haim S, Lebovitz O, Dor J, Orvieto R, Cohen Y (2016). FMR6 may play a role in the pathogenesis of fragile X-associated premature ovarian insufficiency. Gynecological Endocrinology.

[bib60] Emamian ES, Kaytor MD, Duvick LA, Zu T, Tousey SK, Zoghbi HY, Clark HB, Orr HT (2003). Serine 776 of ataxin-1 is critical for polyglutamine-induced disease in SCA1 transgenic mice. Neuron.

[bib61] Entezam A, Biacsi R, Orrison B, Saha T, Hoffman GE, Grabczyk E, Nussbaum RL, Usdin K (2007). Regional FMRP deficits and large repeat expansions into the full mutation range in a new fragile X premutation mouse model. Gene.

[bib62] Fan HY, Cheng KK, Klein HL (1996). Mutations in the RNA polymerase II transcription machinery suppress the hyperrecombination mutant hpr1 delta of *Saccharomyces cerevisiae*. Genetics.

[bib63] Fan HY, Merker RJ, Klein HL (2001). High-Copy-Number expression of Sub2p, a member of the RNA helicase superfamily, suppresses hpr1-mediated genomic instability. Molecular and Cellular Biology.

[bib64] Fardaei M, Larkin K, Brook JD, Hamshere MG (2001). In vivo co-localisation of MBNL protein with DMPK expanded-repeat transcripts. Nucleic Acids Research.

[bib65] Faux N (2012). Single amino acid and trinucleotide repeats: function and evolution. Advances in Experimental Medicine and Biology.

[bib66] Fay MM, Anderson PJ, Ivanov P (2017). ALS/FTD-associated C9orf72 repeat RNA promotes phase transitions in vitro and in cells. Cell Reports.

[bib67] Fondon JW, Garner HR (2004). Molecular origins of rapid and continuous morphological evolution. PNAS.

[bib68] Fotsing SF, Margoliash J, Wang C, Saini S, Yanicky R, Shleizer-Burko S, Goren A, Gymrek M (2019). The impact of short tandem repeat variation on gene expression. Nature Genetics.

[bib69] Franich NR, Hickey MA, Zhu C, Osborne GF, Ali N, Chu T, Bove NH, Lemesre V, Lerner RP, Zeitlin SO, Howland D, Neueder A, Landles C, Bates GP, Chesselet M-F (2019). Phenotype onset in Huntington’s disease knock-in mice is correlated with the incomplete splicing of the mutant huntingtin gene. Journal of Neuroscience Research.

[bib70] Freibaum BD, Lu Y, Lopez-Gonzalez R, Kim NC, Almeida S, Lee K-H, Badders N, Valentine M, Miller BL, Wong PC, Petrucelli L, Kim HJ, Gao F-B, Taylor JP (2015). GGGGCC repeat expansion in c9orf72 compromises nucleocytoplasmic transport. Nature.

[bib71] Fry M, Loeb LA (1994). The fragile X syndrome D (CGG) N nucleotide repeats form a stable tetrahelical structure. PNAS.

[bib72] Fu YH, Kuhl DP, Pizzuti A, Pieretti M, Sutcliffe JS, Richards S, Verkerk AJ, Holden JJ, Fenwick RG, Warren ST (1991). Variation of the CGG repeat at the fragile X site results in genetic instability: resolution of the sherman paradox. Cell.

[bib73] Fu YH, Pizzuti A, Fenwick RG, King J, Rajnarayan S, Dunne PW, Dubel J, Nasser GA, Ashizawa T, de Jong P (1992). An unstable triplet repeat in a gene related to myotonic muscular dystrophy. Science.

[bib74] Gao FB, Richter JD, Cleveland DW (2017). Rethinking unconventional translation in neurodegeneration. Cell.

[bib75] Garg P, Martin-Trujillo A, Rodriguez OL, Gies SJ, Hadelia E, Jadhav B, Jain M, Paten B, Sharp AJ (2021). Pervasive cis effects of variation in copy number of large tandem repeats on local DNA methylation and gene expression. American Journal of Human Genetics.

[bib76] Gaspar C, Jannatipour M, Dion P, Laganière J, Sequeiros J, Brais B, Rouleau GA (2000). CAG tract of MJD-1 may be prone to frameshifts causing polyalanine accumulation. Human Molecular Genetics.

[bib77] Gasset-Rosa F, Chillon-Marinas C, Goginashvili A, Atwal RS, Artates JW, Tabet R, Wheeler VC, Bang AG, Cleveland DW, Lagier-Tourenne C (2017). Polyglutamine-expanded huntingtin exacerbates age-related disruption of nuclear integrity and nucleocytoplasmic transport. Neuron.

[bib78] Gecz J, Gedeon AK, Sutherland GR, Mulley JC (1996). Identification of the gene FMR2, associated with FRAXE mental retardation. Nature Genetics.

[bib79] Gecz J (2000). The FMR2 gene, FRAXE and non-specific X-linked mental retardation: clinical and molecular aspects. Annals of Human Genetics.

[bib80] Gemmell NJ (2021). Repetitive DNA: genomic dark matter matters. Nature Reviews. Genetics.

[bib81] Gendron TF, Bieniek KF, Zhang Y-J, Jansen-West K, Ash PEA, Caulfield T, Daughrity L, Dunmore JH, Castanedes-Casey M, Chew J, Cosio DM, van Blitterswijk M, Lee WC, Rademakers R, Boylan KB, Dickson DW, Petrucelli L (2013). Antisense transcripts of the expanded C9ORF72 hexanucleotide repeat form nuclear RNA foci and undergo repeat-associated non-ATG translation in c9ftd/ALS. Acta Neuropathologica.

[bib82] Gerhardt J, Tomishima MJ, Zaninovic N, Colak D, Yan Z, Zhan Q, Rosenwaks Z, Jaffrey SR, Schildkraut CL (2014). The DNA replication program is altered at the FMR1 locus in fragile X embryonic stem cells. Molecular Cell.

[bib83] Gipson TA, Neueder A, Wexler NS, Bates GP, Housman D (2013). Aberrantly spliced HTT, a new player in Huntington’s disease pathogenesis. RNA Biology.

[bib84] Glineburg MR, Todd PK, Charlet-Berguerand N, Sellier C (2018). Repeat-Associated non-AUG (Ran) translation and other molecular mechanisms in fragile X tremor ataxia syndrome. Brain Research.

[bib85] Glover TW (2006). Common fragile sites. Cancer Letters.

[bib86] Gohel D, Sripada L, Prajapati P, Singh K, Roy M, Kotadia D, Tassone F, Charlet-Berguerand N, Singh R (2019). FMRpolyG alters mitochondrial transcripts level and respiratory chain complex assembly in fragile X associated tremor/ataxia syndrome. Biochimica et Biophysica Acta. Molecular Basis of Disease.

[bib87] Goodwin M, Mohan A, Batra R, Lee K-Y, Charizanis K, Fernández Gómez FJ, Eddarkaoui S, Sergeant N, Buée L, Kimura T, Clark HB, Dalton J, Takamura K, Weyn-Vanhentenryck SM, Zhang C, Reid T, Ranum LPW, Day JW, Swanson MS (2015). Mbnl sequestration by toxic RNAs and RNA misprocessing in the myotonic dystrophy brain. Cell Reports.

[bib88] Grabczyk E, Fishman MC (1995). A long purine-pyrimidine homopolymer acts as a transcriptional diode. The Journal of Biological Chemistry.

[bib89] Greene E, Mahishi L, Entezam A, Kumari D, Usdin K (2007). Repeat-Induced epigenetic changes in intron 1 of the frataxin gene and its consequences in Friedreich ataxia. Nucleic Acids Research.

[bib90] Grima JC, Daigle JG, Arbez N, Cunningham KC, Zhang K, Ochaba J, Geater C, Morozko E, Stocksdale J, Glatzer JC, Pham JT, Ahmed I, Peng Q, Wadhwa H, Pletnikova O, Troncoso JC, Duan W, Snyder SH, Ranum LPW, Thompson LM, Lloyd TE, Ross CA, Rothstein JD (2017). Mutant huntingtin disrupts the nuclear pore complex. Neuron.

[bib91] Groh M, Silva LM, Gromak N (2014). Mechanisms of transcriptional dysregulation in repeat expansion disorders. Biochemical Society Transactions.

[bib92] Gu Y, Shen Y, Gibbs RA, Nelson DL (1996). Identification of FMR2, a novel gene associated with the FRAXE CCG repeat and CpG island. Nature Genetics.

[bib93] Gymrek M, Willems T, Guilmatre A, Zeng H, Markus B, Georgiev S, Daly MJ, Price AL, Pritchard JK, Sharp AJ, Erlich Y (2016). Abundant contribution of short tandem repeats to gene expression variation in humans. Nature Genetics.

[bib94] Gymrek M (2017). A genomic view of short tandem repeats. Current Opinion in Genetics & Development.

[bib95] Gymrek M, Willems T, Reich D, Erlich Y (2017). Interpreting short tandem repeat variations in humans using mutational constraint. Nature Genetics.

[bib96] Hagerman RJ, Jackson AW, Levitas A, Rimland B, Braden M (1986). An analysis of autism in fifty males with the fragile X syndrome. American Journal of Medical Genetics.

[bib97] Hagerman RJ, Leehey M, Heinrichs W, Tassone F, Wilson R, Hills J, Grigsby J, Gage B, Hagerman PJ (2001). Intention tremor, parkinsonism, and generalized brain atrophy in male carriers of fragile X. Neurology.

[bib98] Hagerman RJ, Hagerman PJ (2002a). Fragile X Syndrome: Diagnosis, Treatment, and Research.

[bib99] Hagerman RJ, Hagerman PJ (2002b). The fragile X premutation: into the phenotypic fold. Current Opinion in Genetics & Development.

[bib100] Hagerman PJ, Hagerman RJ (2004). Fragile X-associated Tremor/Ataxia Syndrome (FXTAS). Mental Retardation and Developmental Disabilities Research Reviews.

[bib101] Hagerman PJ, Hagerman RJ (2015). Fragile X-associated tremor/ataxia syndrome. Annals of the New York Academy of Sciences.

[bib102] Hagerman RJ, Berry-Kravis E, Hazlett HC, Bailey DB, Moine H, Kooy RF, Tassone F, Gantois I, Sonenberg N, Mandel JL, Hagerman PJ (2017). Fragile X syndrome. Nature Reviews. Disease Primers.

[bib103] Hammock EAD, Young LJ (2005). Microsatellite instability generates diversity in brain and sociobehavioral traits. Science.

[bib104] Hannan AJ (2018). Tandem repeats mediating genetic plasticity in health and disease. Nature Reviews. Genetics.

[bib105] Hansen RS, Gartler SM, Scott CR, Chen SH, Laird CD (1992). Methylation analysis of CGG sites in the CpG island of the human FMR1 gene. Human Molecular Genetics.

[bib106] Harley HG, Brook JD, Rundle SA, Crow S, Reardon W, Buckler AJ, Harper PS, Housman DE, Shaw DJ (1992). Expansion of an unstable DNA region and phenotypic variation in myotonic dystrophy. Nature.

[bib107] Hautbergue GM, Castelli LM, Ferraiuolo L, Sanchez-Martinez A, Cooper-Knock J, Higginbottom A, Lin YH, Bauer CS, Dodd JE, Myszczynska MA, Alam SM, Garneret P, Chandran JS, Karyka E, Stopford MJ, Smith EF, Kirby J, Meyer K, Kaspar BK, Isaacs AM, El-Khamisy SF, De Vos KJ, Ning K, Azzouz M, Whitworth AJ, Shaw PJ (2017). SRSF1-dependent nuclear export inhibition of c9orf72 repeat transcripts prevents neurodegeneration and associated motor deficits. Nature Communications.

[bib108] He F, Krans A, Freibaum BD, Taylor JP, Todd PK (2014). Tdp-43 suppresses CGG repeat-induced neurotoxicity through interactions with hnRNP A2/B1. Human Molecular Genetics.

[bib109] Heitz D, Rousseau F, Devys D, Saccone S, Abderrahim H, Le Paslier D, Cohen D, Vincent A, Toniolo D, Della Valle G (1991). Isolation of sequences that span the fragile X and identification of a fragile X-related cpg island. Science.

[bib110] Herman D, Jenssen K, Burnett R, Soragni E, Perlman SL, Gottesfeld JM (2006). Histone deacetylase inhibitors reverse gene silencing in Friedreich’s ataxia. Nature Chemical Biology.

[bib111] Hinnebusch AG, Ivanov IP, Sonenberg N (2016). Translational control by 5’-untranslated regions of eukaryotic mrnas. Science.

[bib112] Hoem G, Bowitz Larsen K, Øvervatn A, Brech A, Lamark T, Sjøttem E, Johansen T (2019). The fmrpolyglycine protein mediates aggregate formation and toxicity independent of the CGG mrna hairpin in a cellular model for FXTAS. Frontiers in Genetics.

[bib113] Huh GS, Hynes RO (1994). Regulation of alternative pre-mRNA splicing by a novel repeated hexanucleotide element. Genes & Development.

[bib114] Irwin S, Vandelft M, Pinchev D, Howell JL, Graczyk J, Orr HT, Truant R (2005). RNA association and nucleocytoplasmic shuttling by ataxin-1. Journal of Cell Science.

[bib115] Ishiguro T, Sato N, Ueyama M, Fujikake N, Sellier C, Kanegami A, Tokuda E, Zamiri B, Gall-Duncan T, Mirceta M, Furukawa Y, Yokota T, Wada K, Taylor JP, Pearson CE, Charlet-Berguerand N, Mizusawa H, Nagai Y, Ishikawa K (2017). Regulatory role of RNA chaperone TDP-43 for RNA misfolding and repeat-associated translation in SCA31. Neuron.

[bib116] Ishiura H, Doi K, Mitsui J, Yoshimura J, Matsukawa MK, Fujiyama A, Toyoshima Y, Kakita A, Takahashi H, Suzuki Y, Sugano S, Qu W, Ichikawa K, Yurino H, Higasa K, Shibata S, Mitsue A, Tanaka M, Ichikawa Y, Takahashi Y, Date H, Matsukawa T, Kanda J, Nakamoto FK, Higashihara M, Abe K, Koike R, Sasagawa M, Kuroha Y, Hasegawa N, Kanesawa N, Kondo T, Hitomi T, Tada M, Takano H, Saito Y, Sanpei K, Onodera O, Nishizawa M, Nakamura M, Yasuda T, Sakiyama Y, Otsuka M, Ueki A, Kaida K-I, Shimizu J, Hanajima R, Hayashi T, Terao Y, Inomata-Terada S, Hamada M, Shirota Y, Kubota A, Ugawa Y, Koh K, Takiyama Y, Ohsawa-Yoshida N, Ishiura S, Yamasaki R, Tamaoka A, Akiyama H, Otsuki T, Sano A, Ikeda A, Goto J, Morishita S, Tsuji S (2018). Expansions of intronic TTTCA and TTTTA repeats in benign adult familial myoclonic epilepsy. Nature Genetics.

[bib117] Ishiura H, Shibata S, Yoshimura J, Suzuki Y, Qu W, Doi K, Almansour MA, Kikuchi JK, Taira M, Mitsui J, Takahashi Y, Ichikawa Y, Mano T, Iwata A, Harigaya Y, Matsukawa MK, Matsukawa T, Tanaka M, Shirota Y, Ohtomo R, Kowa H, Date H, Mitsue A, Hatsuta H, Morimoto S, Murayama S, Shiio Y, Saito Y, Mitsutake A, Kawai M, Sasaki T, Sugiyama Y, Hamada M, Ohtomo G, Terao Y, Nakazato Y, Takeda A, Sakiyama Y, Umeda-Kameyama Y, Shinmi J, Ogata K, Kohno Y, Lim S-Y, Tan AH, Shimizu J, Goto J, Nishino I, Toda T, Morishita S, Tsuji S (2019). Noncoding CGG repeat expansions in neuronal intranuclear inclusion disease, oculopharyngodistal myopathy and an overlapping disease. Nature Genetics.

[bib118] Iwahashi CK, Yasui DH, An H-J, Greco CM, Tassone F, Nannen K, Babineau B, Lebrilla CB, Hagerman RJ, Hagerman PJ (2006). Protein composition of the intranuclear inclusions of FXTAS. Brain.

[bib119] Jacquemont S, Hagerman RJ, Leehey M, Grigsby J, Zhang L, Brunberg JA, Greco C, Des Portes V, Jardini T, Levine R, Berry-Kravis E, Brown WT, Schaeffer S, Kissel J, Tassone F, Hagerman PJ (2003). Fragile X premutation tremor/ataxia syndrome: molecular, clinical, and neuroimaging correlates. American Journal of Human Genetics.

[bib120] Jain A, Vale RD (2017). Rna phase transitions in repeat expansion disorders. Nature.

[bib121] Jakubosky D, D’Antonio M, Bonder MJ, Smail C, Donovan MKR, Young Greenwald WW, Matsui H, D’Antonio-Chronowska A, Stegle O, Smith EN, Montgomery SB, DeBoever C, Frazer KA, i2QTL Consortium (2020). Properties of structural variants and short tandem repeats associated with gene expression and complex traits. Nature Communications.

[bib122] Janitz K, Janitz M, Tollefsbol T (2011). Handbook of Epigenetics.

[bib123] Jiang H, Mankodi A, Swanson MS, Moxley RT, Thornton CA (2004). Myotonic dystrophy type 1 is associated with nuclear foci of mutant RNA, sequestration of muscleblind proteins and deregulated alternative splicing in neurons. Human Molecular Genetics.

[bib124] Jimeno S, Rondón AG, Luna R, Aguilera A (2002). The yeast THO complex and mRNA export factors link RNA metabolism with transcription and genome instability. The EMBO Journal.

[bib125] Jin P, Duan R, Qurashi A, Qin Y, Tian D, Rosser TC, Liu H, Feng Y, Warren ST (2007). Pur alpha binds to rCGG repeats and modulates repeat-mediated neurodegeneration in a *Drosophila* model of fragile X tremor/ataxia syndrome. Neuron.

[bib126] Jog SP, Paul S, Dansithong W, Tring S, Comai L, Reddy S (2012). Rna splicing is responsive to MBNL1 dose. PLOS ONE.

[bib127] Jovičić A, Mertens J, Boeynaems S, Bogaert E, Chai N, Yamada SB, Paul JW, Sun S, Herdy JR, Bieri G, Kramer NJ, Gage FH, Van Den Bosch L, Robberecht W, Gitler AD (2015). Modifiers of c9orf72 dipeptide repeat toxicity connect nucleocytoplasmic transport defects to FTD/ALS. Nature Neuroscience.

[bib128] Kajava AV (2012). Tandem repeats in proteins: from sequence to structure. Journal of Structural Biology.

[bib129] Katsuno M., Adachi H, Kume A, Li M, Nakagomi Y, Niwa H, Sang C, Kobayashi Y, Doyu M, Sobue G (2002). Testosterone reduction prevents phenotypic expression in a transgenic mouse model of spinal and bulbar muscular atrophy. Neuron.

[bib130] Katsuno M, Adachi H, Minamiyama M, Waza M, Tokui K, Banno H, Suzuki K, Onoda Y, Tanaka F, Doyu M, Sobue G (2006). Reversible disruption of dynactin 1-mediated retrograde axonal transport in polyglutamine-induced motor neuron degeneration. The Journal of Neuroscience.

[bib131] Kearse MG, Todd PK (2014). Repeat-Associated non-AUG translation and its impact in neurodegenerative disease. Neurotherapeutics.

[bib132] Kearse MG, Green KM, Krans A, Rodriguez CM, Linsalata AE, Goldstrohm AC, Todd PK (2016). Cgg repeat-associated non-AUG translation utilizes a cap-dependent scanning mechanism of initiation to produce toxic proteins. Molecular Cell.

[bib133] Kenneson A, Zhang F, Hagedorn CH, Warren ST (2001). Reduced FMRP and increased FMR1 transcription is proportionally associated with CGG repeat number in intermediate-length and premutation carriers. Human Molecular Genetics.

[bib134] Kettani A, Kumar RA, Patel DJ (1995). Solution structure of a DNA quadruplex containing the fragile X syndrome triplet repeat. Journal of Molecular Biology.

[bib135] Khalil AM, Faghihi MA, Modarresi F, Brothers SP, Wahlestedt C, Bajic V (2008). A novel RNA transcript with antiapoptotic function is silenced in fragile X syndrome. PLOS ONE.

[bib136] Kim M, Ahn SH, Krogan NJ, Greenblatt JF, Buratowski S (2004). Transitions in RNA polymerase II elongation complexes at the 3’ ends of genes. The EMBO Journal.

[bib137] Klement IA, Skinner PJ, Kaytor MD, Yi H, Hersch SM, Clark HB, Zoghbi HY, Orr HT (1998). Ataxin-1 nuclear localization and aggregation: role in polyglutamine-induced disease in SCA1 transgenic mice. Cell.

[bib138] Klusek J, Porter A, Abbeduto L, Adayev T, Tassone F, Mailick MR, Glicksman A, Tonnsen BL, Roberts JE (2018). Curvilinear association between language disfluency and *fmr1* CGG repeat size across the normal, intermediate, and premutation range. Frontiers in Genetics.

[bib139] Knight SJL, Flannery AV, Hirst MC, Campbell L, Christodoulou Z, Phelps SR, Pointon J, Middleton-Price HR, Barnicoat A, Pembrey ME (1993). Trinucleotide repeat amplification and hypermethylation of a CpG island in FRAXE mental retardation. Cell.

[bib140] Korade-Mirnics Z, Babitzke P, Hoffman E (1998). Myotonic dystrophy: molecular windows on a complex etiology. Nucleic Acids Research.

[bib141] Kozak M (1980). Influence of mRNA secondary structure on binding and migration of 40S ribosomal subunits. Cell.

[bib142] Kozak M (1986). Influences of mrna secondary structure on initiation by eukaryotic ribosomes. PNAS.

[bib143] Krans A, Skariah G, Zhang Y, Bayly B, Todd PK (2019). Neuropathology of ran translation proteins in fragile X-associated tremor/ataxia syndrome. Acta Neuropathologica Communications.

[bib144] Krzyzosiak WJ, Sobczak K, Wojciechowska M, Fiszer A, Mykowska A, Kozlowski P (2012). Triplet repeat RNA structure and its role as pathogenic agent and therapeutic target. Nucleic Acids Research.

[bib145] Kudla G, Murray AW, Tollervey D, Plotkin JB (2009). Coding-Sequence determinants of gene expression in *Escherichia coli*. Science.

[bib146] La Spada AR, Roling DB, Harding AE, Warner CL, Spiegel R, Hausmanowa-Petrusewicz I, Yee WC, Fischbeck KH (1992). Meiotic stability and genotype-phenotype correlation of the trinucleotide repeat in X-linked spinal and bulbar muscular atrophy. Nature Genetics.

[bib147] Ladd PD, Smith LE, Rabaia NA, Moore JM, Georges SA, Hansen RS, Hagerman RJ, Tassone F, Tapscott SJ, Filippova GN (2007). An antisense transcript spanning the CGG repeat region of FMR1 is upregulated in premutation carriers but silenced in full mutation individuals. Human Molecular Genetics.

[bib148] Lai S, O’Callaghan B, Zoghbi HY, Orr HT (2011). 14-3-3 binding to ataxin-1 (ATXN1) regulates its dephosphorylation at Ser-776 and transport to the nucleus. The Journal of Biological Chemistry.

[bib149] Lam YC, Bowman AB, Jafar-Nejad P, Lim J, Richman R, Fryer JD, Hyun ED, Duvick LA, Orr HT, Botas J, Zoghbi HY (2006). Ataxin-1 interacts with the repressor capicua in its native complex to cause SCA1 neuropathology. Cell.

[bib150] Lander ES, Linton LM, Birren B, Nusbaum C, Zody MC, Baldwin J, Devon K, Dewar K, Doyle M, FitzHugh W, Funke R, Gage D, Harris K, Heaford A, Howland J, Kann L, Lehoczky J, LeVine R, McEwan P, McKernan K, Meldrim J, Mesirov JP, Miranda C, Morris W, Naylor J, Raymond C, Rosetti M, Santos R, Sheridan A, Sougnez C, Stange-Thomann Y, Stojanovic N, Subramanian A, Wyman D, Rogers J, Sulston J, Ainscough R, Beck S, Bentley D, Burton J, Clee C, Carter N, Coulson A, Deadman R, Deloukas P, Dunham A, Dunham I, Durbin R, French L, Grafham D, Gregory S, Hubbard T, Humphray S, Hunt A, Jones M, Lloyd C, McMurray A, Matthews L, Mercer S, Milne S, Mullikin JC, Mungall A, Plumb R, Ross M, Shownkeen R, Sims S, Waterston RH, Wilson RK, Hillier LW, McPherson JD, Marra MA, Mardis ER, Fulton LA, Chinwalla AT, Pepin KH, Gish WR, Chissoe SL, Wendl MC, Delehaunty KD, Miner TL, Delehaunty A, Kramer JB, Cook LL, Fulton RS, Johnson DL, Minx PJ, Clifton SW, Hawkins T, Branscomb E, Predki P, Richardson P, Wenning S, Slezak T, Doggett N, Cheng JF, Olsen A, Lucas S, Elkin C, Uberbacher E, Frazier M, Gibbs RA, Muzny DM, Scherer SE, Bouck JB, Sodergren EJ, Worley KC, Rives CM, Gorrell JH, Metzker ML, Naylor SL, Kucherlapati RS, Nelson DL, Weinstock GM, Sakaki Y, Fujiyama A, Hattori M, Yada T, Toyoda A, Itoh T, Kawagoe C, Watanabe H, Totoki Y, Taylor T, Weissenbach J, Heilig R, Saurin W, Artiguenave F, Brottier P, Bruls T, Pelletier E, Robert C, Wincker P, Smith DR, Doucette-Stamm L, Rubenfield M, Weinstock K, Lee HM, Dubois J, Rosenthal A, Platzer M, Nyakatura G, Taudien S, Rump A, Yang H, Yu J, Wang J, Huang G, Gu J, Hood L, Rowen L, Madan A, Qin S, Davis RW, Federspiel NA, Abola AP, Proctor MJ, Myers RM, Schmutz J, Dickson M, Grimwood J, Cox DR, Olson MV, Kaul R, Raymond C, Shimizu N, Kawasaki K, Minoshima S, Evans GA, Athanasiou M, Schultz R, Roe BA, Chen F, Pan H, Ramser J, Lehrach H, Reinhardt R, McCombie WR, de la Bastide M, Dedhia N, Blöcker H, Hornischer K, Nordsiek G, Agarwala R, Aravind L, Bailey JA, Bateman A, Batzoglou S, Birney E, Bork P, Brown DG, Burge CB, Cerutti L, Chen HC, Church D, Clamp M, Copley RR, Doerks T, Eddy SR, Eichler EE, Furey TS, Galagan J, Gilbert JG, Harmon C, Hayashizaki Y, Haussler D, Hermjakob H, Hokamp K, Jang W, Johnson LS, Jones TA, Kasif S, Kaspryzk A, Kennedy S, Kent WJ, Kitts P, Koonin EV, Korf I, Kulp D, Lancet D, Lowe TM, McLysaght A, Mikkelsen T, Moran JV, Mulder N, Pollara VJ, Ponting CP, Schuler G, Schultz J, Slater G, Smit AF, Stupka E, Szustakowki J, Thierry-Mieg D, Thierry-Mieg J, Wagner L, Wallis J, Wheeler R, Williams A, Wolf YI, Wolfe KH, Yang SP, Yeh RF, Collins F, Guyer MS, Peterson J, Felsenfeld A, Wetterstrand KA, Patrinos A, Morgan MJ, de Jong P, Catanese JJ, Osoegawa K, Shizuya H, Choi S, Chen YJ, Szustakowki J, International Human Genome Sequencing Consortium (2001). Initial sequencing and analysis of the human genome. Nature.

[bib151] Leehey MA, Munhoz RP, Lang AE, Brunberg JA, Grigsby J, Greco C, Jacquemont S, Tassone F, Lozano AM, Hagerman PJ, Hagerman RJ (2003). The fragile X premutation presenting as essential tremor. Archives of Neurology.

[bib152] Levdansky E, Romano J, Shadkchan Y, Sharon H, Verstrepen KJ, Fink GR, Osherov N (2007). Coding tandem repeats generate diversity in Aspergillus fumigatus genes. Eukaryotic Cell.

[bib153] Li JJ, Chew GL, Biggin MD (2017). Quantitating translational control: mRNA abundance-dependent and independent contributions and the mRNA sequences that specify them. Nucleic Acids Research.

[bib154] Lian Y, Garner HR (2005). Evidence for the regulation of alternative splicing via complementary DNA sequence repeats. Bioinformatics.

[bib155] Lim LP, Sharp PA (1998). Alternative splicing of the fibronectin EIIIB exon depends on specific TGCATG repeats. Molecular and Cellular Biology.

[bib156] Liquori CL, Ricker K, Moseley ML, Jacobsen JF, Kress W, Naylor SL, Day JW, Ranum LPW (2001). Myotonic dystrophy type 2 caused by a CCTG expansion in intron 1 of ZNF9. Science.

[bib157] Liquori CL, Ikeda Y, Weatherspoon M, Ricker K, Schoser BGH, Dalton JC, Day JW, Ranum LPW (2003). Myotonic dystrophy type 2: human founder haplotype and evolutionary conservation of the repeat tract. American Journal of Human Genetics.

[bib158] Liu Q, Zhang K, Kang Y, Li Y, Deng P, Li Y, Tian Y, Sun Q, Tang Y, Xu K, Zhou Y, Wang J-L, Guo J, Li J-D, Xia K, Meng Q, Allen EG, Wen Z, Li Z, Jiang H, Shen L, Duan R, Yao B, Tang B, Jin P, Pan Y (2022). Expression of expanded GGC repeats within NOTCH2NLC causes behavioral deficits and neurodegeneration in a mouse model of neuronal intranuclear inclusion disease. Science Advances.

[bib159] López Castel A, Nakamori M, Tomé S, Chitayat D, Gourdon G, Thornton CA, Pearson CE (2011). Expanded CTG repeat demarcates a boundary for abnormal CpG methylation in myotonic dystrophy patient tissues. Human Molecular Genetics.

[bib160] López-Martínez A, Soblechero-Martín P, de-la-Puente-Ovejero L, Nogales-Gadea G, Arechavala-Gomeza V (2020). An overview of alternative splicing defects implicated in myotonic dystrophy type I. Genes.

[bib161] Lu JY, Chang L, Li T, Wang T, Yin Y, Zhan G, Han X, Zhang K, Tao Y, Percharde M, Wang L, Peng Q, Yan P, Zhang H, Bi X, Shao W, Hong Y, Wu Z, Ma R, Wang P, Li W, Zhang J, Chang Z, Hou Y, Zhu B, Ramalho-Santos M, Li P, Xie W, Na J, Sun Y, Shen X (2021). Homotypic clustering of L1 and B1/alu repeats compartmentalizes the 3D genome. Cell Research.

[bib162] Lubs HA (1969). A marker X chromosome. American Journal of Human Genetics.

[bib163] Ma L, Herren AW, Espinal G, Randol J, McLaughlin B, Martinez-Cerdeño V, Pessah IN, Hagerman RJ, Hagerman PJ (2019). Composition of the intranuclear inclusions of fragile X-associated tremor/ataxia syndrome. Acta Neuropathologica Communications.

[bib164] MacDonald ME, Ambrose CM, Duyao MP, Myers RH, Lin C, Srinidhi L, Barnes G, Taylor SA, James M, Groot N, MacFarlane H, Jenkins B, Anderson MA, Wexler NS, Gusella JF, Bates GP, Baxendale S, Hummerich H, Kirby S, North M, Youngman S, Mott R, Zehetner G, Sedlacek Z, Poustka A, Frischauf AM, Lehrach H, Buckler AJ, Church D, Doucette-Stamm L, O’Donovan MC, Riba-Ramirez L, Shah M, Stanton VP, Strobel SA, Draths KM, Wales JL, Dervan P, Housman DE, Altherr M, Shiang R, Thompson L, Fielder T, Wasmuth JJ, Tagle D, Valdes J, Elmer L, Allard M, Castilla L, Swaroop M, Blanchard K, Collins FS, Snell R, Holloway T, Gillespie K, Datson N, Shaw D, Harper PS (1993). A novel gene containing A trinucleotide repeat that is expanded and unstable on huntington’s disease chromosomes: the huntington’s disease collaborative research group. Cell.

[bib165] Mahadevan M, Tsilfidis C, Sabourin L, Shutler G, Amemiya C, Jansen G, Neville C, Narang M, Barceló J, O’Hoy K (1992). Myotonic dystrophy mutation: an unstable CTG repeat in the 3’ untranslated region of the gene. Science.

[bib166] Mailick MR, Hong J, Rathouz P, Baker MW, Greenberg JS, Smith L, Maenner M (2014). Low-normal FMR1 CGG repeat length: phenotypic associations. Frontiers in Genetics.

[bib167] Malgowska M, Gudanis D, Kierzek R, Wyszko E, Gabelica V, Gdaniec Z (2014). Distinctive structural motifs of RNA G-quadruplexes composed of AGG, CGG and UGG trinucleotide repeats. Nucleic Acids Research.

[bib168] Malik I, Kelley CP, Wang ET, Todd PK (2021a). Molecular mechanisms underlying nucleotide repeat expansion disorders. Nature Reviews. Molecular Cell Biology.

[bib169] Malik I, Tseng YJ, Wright SE, Zheng K, Ramaiyer P, Green KM, Todd PK (2021b). SRSF protein kinase 1 modulates RAN translation and suppresses CGG repeat toxicity. EMBO Molecular Medicine.

[bib170] Mallick S, Li H, Lipson M, Mathieson I, Gymrek M, Racimo F, Zhao M, Chennagiri N, Nordenfelt S, Tandon A, Skoglund P, Lazaridis I, Sankararaman S, Fu Q, Rohland N, Renaud G, Erlich Y, Willems T, Gallo C, Spence JP, Song YS, Poletti G, Balloux F, van Driem G, de Knijff P, Romero IG, Jha AR, Behar DM, Bravi CM, Capelli C, Hervig T, Moreno-Estrada A, Posukh OL, Balanovska E, Balanovsky O, Karachanak-Yankova S, Sahakyan H, Toncheva D, Yepiskoposyan L, Tyler-Smith C, Xue Y, Abdullah MS, Ruiz-Linares A, Beall CM, Di Rienzo A, Jeong C, Starikovskaya EB, Metspalu E, Parik J, Villems R, Henn BM, Hodoglugil U, Mahley R, Sajantila A, Stamatoyannopoulos G, Wee JTS, Khusainova R, Khusnutdinova E, Litvinov S, Ayodo G, Comas D, Hammer MF, Kivisild T, Klitz W, Winkler CA, Labuda D, Bamshad M, Jorde LB, Tishkoff SA, Watkins WS, Metspalu M, Dryomov S, Sukernik R, Singh L, Thangaraj K, Pääbo S, Kelso J, Patterson N, Reich D (2016). The simons genome diversity project: 300 genomes from 142 diverse populations. Nature.

[bib171] Maltby CJ, Schofield JPR, Houghton SD, O’Kelly I, Vargas-Caballero M, Deinhardt K, Coldwell MJ (2020). A 5′ UTR Ggn repeat controls localisation and translation of a potassium leak channel mRNA through G-quadruplex formation. Nucleic Acids Research.

[bib172] Mankodi A, Urbinati CR, Yuan QP, Moxley RT, Sansone V, Krym M, Henderson D, Schalling M, Swanson MS, Thornton CA (2001). Muscleblind localizes to nuclear foci of aberrant RNA in myotonic dystrophy types 1 and 2. Human Molecular Genetics.

[bib173] Marcotte EM, Pellegrini M, Yeates TO, Eisenberg D (1999). A census of protein repeats. Journal of Molecular Biology.

[bib174] Martin JP, Bell J (1943). A pedigree of mental defect showing sex-linkage. Journal of Neurology and Psychiatry.

[bib175] Mastroyiannopoulos NP, Shammas C, Phylactou LA (2010). Tackling the pathogenesis of RNA nuclear retention in myotonic dystrophy. Biology of the Cell.

[bib176] May S, Hornburg D, Schludi MH, Arzberger T, Rentzsch K, Schwenk BM, Grässer FA, Mori K, Kremmer E, Banzhaf-Strathmann J, Mann M, Meissner F, Edbauer D (2014). C9Orf72 FTLD/ALS-associated Gly-Ala dipeptide repeat proteins cause neuronal toxicity and UNC119 sequestration. Acta Neuropathologica.

[bib177] McClintock B (1950). The origin and behavior of mutable loci in maize. PNAS.

[bib178] McConkie-Rosell A, Lachiewicz AM, Spiridigliozzi GA, Tarleton J, Schoenwald S, Phelan MC, Goonewardena P, Ding X, Brown WT (1993). Evidence that methylation of the FMR-I locus is responsible for variable phenotypic expression of the fragile X syndrome. American Journal of Human Genetics.

[bib179] McEachin ZT, Gendron TF, Raj N, García-Murias M, Banerjee A, Purcell RH, Ward PJ, Todd TW, Merritt-Garza ME, Jansen-West K, Hales CM, García-Sobrino T, Quintáns B, Holler CJ, Taylor G, San Millán B, Teijeira S, Yamashita T, Ohkubo R, Boulis NM, Xu C, Wen Z, Streichenberger N, Fogel BL, Kukar T, Abe K, Dickson DW, Arias M, Glass JD, Jiang J, Tansey MG, Sobrido M-J, Petrucelli L, Rossoll W, Bassell GJ, Neuro–CEB Neuropathology Network (2020). Chimeric peptide species contribute to divergent dipeptide repeat pathology in c9als/FTD and SCA36. Neuron.

[bib180] McIver LJ, Fondon JW, Skinner MA, Garner HR (2011). Evaluation of microsatellite variation in the 1000 genomes project pilot studies is indicative of the quality and utility of the RAW data and alignments. Genomics.

[bib181] McIver LJ, McCormick JF, Martin A, Fondon JW, Garner HR (2013). Population-scale analysis of human microsatellites reveals novel sources of exonic variation. Gene.

[bib182] Menzies FM, Huebener J, Renna M, Bonin M, Riess O, Rubinsztein DC (2010). Autophagy induction reduces mutant ataxin-3 levels and toxicity in a mouse model of spinocerebellar ataxia type 3. Brain.

[bib183] Miller JW, Urbinati CR, Teng-Umnuay P, Stenberg MG, Byrne BJ, Thornton CA, Swanson MS (2000). Recruitment of human muscleblind proteins to (CUG) (N) expansions associated with myotonic dystrophy. The EMBO Journal.

[bib184] Millette MM, Holland ED, Tenpas TJ, Dent EW (2022). A single transcript knockdown-replacement strategy employing 5’ UTR secondary structures to precisely titrate rescue protein translation. Frontiers in Genome Editing.

[bib185] Mitra I, Huang B, Mousavi N, Ma N, Lamkin M, Yanicky R, Shleizer-Burko S, Lohmueller KE, Gymrek M (2021). Patterns of de novo tandem repeat mutations and their role in autism. Nature.

[bib186] Mitsuhashi S, Matsumoto N (2020). Long-Read sequencing for rare human genetic diseases. Journal of Human Genetics.

[bib187] Mizielinska S, Grönke S, Niccoli T, Ridler CE, Clayton EL, Devoy A, Moens T, Norona FE, Woollacott IOC, Pietrzyk J, Cleverley K, Nicoll AJ, Pickering-Brown S, Dols J, Cabecinha M, Hendrich O, Fratta P, Fisher EMC, Partridge L, Isaacs AM (2014). C9Orf72 repeat expansions cause neurodegeneration in *Drosophila* through arginine-rich proteins. Science.

[bib188] Mojarad BA, Engchuan W, Trost B, Backstrom I, Yin Y, Thiruvahindrapuram B, Pallotto L, Mitina A, Khan M, Pellecchia G, Haque B, Guo K, Heung T, Costain G, Scherer SW, Marshall CR, Pearson CE, Bassett AS, Yuen RKC (2022). Genome-Wide tandem repeat expansions contribute to schizophrenia risk. Molecular Psychiatry.

[bib189] Montie HL, Cho MS, Holder L, Liu Y, Tsvetkov AS, Finkbeiner S, Merry DE (2009). Cytoplasmic retention of polyglutamine-expanded androgen receptor ameliorates disease via autophagy in a mouse model of spinal and bulbar muscular atrophy. Human Molecular Genetics.

[bib190] Mori K, Arzberger T, Grässer FA, Gijselinck I, May S, Rentzsch K, Weng SM, Schludi MH, van der Zee J, Cruts M, Van Broeckhoven C, Kremmer E, Kretzschmar HA, Haass C, Edbauer D (2013a). Bidirectional transcripts of the expanded c9orf72 hexanucleotide repeat are translated into aggregating dipeptide repeat proteins. Acta Neuropathologica.

[bib191] Mori K, Weng SM, Arzberger T, May S, Rentzsch K, Kremmer E, Schmid B, Kretzschmar HA, Cruts M, Van Broeckhoven C, Haass C, Edbauer D (2013b). The c9orf72 GGGGCC repeat is translated into aggregating dipeptide-repeat proteins in FTLD/ALS. Science.

[bib192] Moseley ML, Zu T, Ikeda Y, Gao W, Mosemiller AK, Daughters RS, Chen G, Weatherspoon MR, Clark HB, Ebner TJ, Day JW, Ranum LPW (2006). Bidirectional expression of CUG and CAG expansion transcripts and intranuclear polyglutamine inclusions in spinocerebellar ataxia type 8. Nature Genetics.

[bib193] Mulley JC, Yu S, Loesch DZ, Hay DA, Donnelly A, Gedeon AK, Carbonell P, López I, Glover G, Gabarrón I (1995). Fraxe and mental retardation. Journal of Medical Genetics.

[bib194] Muro AF, Caputi M, Pariyarath R, Pagani F, Buratti E, Baralle FE (1999). Regulation of fibronectin EDA exon alternative splicing: possible role of RNA secondary structure for enhancer display. Molecular and Cellular Biology.

[bib195] Murray A, Ennis S, MacSwiney F, Webb J, Morton NE (2000a). Reproductive and menstrual history of females with fragile X expansions. European Journal of Human Genetics.

[bib196] Murray A, Ennis S, Morton N (2000b). No evidence for parent of origin influencing premature ovarian failure in fragile X premutation carriers. American Journal of Human Genetics.

[bib197] Muslimov IA, Patel MV, Rose A, Tiedge H (2011). Spatial code recognition in neuronal RNA targeting: role of RNA-hnrnp A2 interactions. The Journal of Cell Biology.

[bib198] Mykowska A, Sobczak K, Wojciechowska M, Kozlowski P, Krzyzosiak WJ (2011). Cag repeats mimic CUG repeats in the misregulation of alternative splicing. Nucleic Acids Research.

[bib199] Myrick LK, Nakamoto-Kinoshita M, Lindor NM, Kirmani S, Cheng X, Warren ST (2014). Fragile X syndrome due to a missense mutation. European Journal of Human Genetics.

[bib200] Nakamura H, Doi H, Mitsuhashi S, Miyatake S, Katoh K, Frith MC, Asano T, Kudo Y, Ikeda T, Kubota S, Kunii M, Kitazawa Y, Tada M, Okamoto M, Joki H, Takeuchi H, Matsumoto N, Tanaka F (2020). Long-Read sequencing identifies the pathogenic nucleotide repeat expansion in RFC1 in a Japanese case of canvas. Journal of Human Genetics.

[bib201] Nasrallah MP, Cho G, Simonet JC, Putt ME, Kitamura K, Golden JA (2012). Differential effects of a polyalanine tract expansion in Arx on neural development and gene expression. Human Molecular Genetics.

[bib202] Neueder A, Landles C, Ghosh R, Howland D, Myers RH, Faull RLM, Tabrizi SJ, Bates GP (2017). The pathogenic exon 1 HTT protein is produced by incomplete splicing in huntington’s disease patients. Scientific Reports.

[bib203] Neueder A, Dumas AA, Benjamin AC, Bates GP (2018). Regulatory mechanisms of incomplete huntingtin mrna splicing. Nature Communications.

[bib204] Niederer RO, Rojas-Duran MF, Zinshteyn B, Gilbert WV (2022). Direct analysis of ribosome targeting illuminates thousand-fold regulation of translation initiation. Cell Systems.

[bib205] Nikumbh S, Pfeifer N (2017). Genetic sequence-based prediction of long-range chromatin interactions suggests a potential role of short tandem repeat sequences in genome organization. BMC Bioinformatics.

[bib206] Oberlé I, Rousseau F, Heitz D, Kretz C, Devys D, Hanauer A, Boué J, Bertheas MF, Mandel JL (1991). Instability of a 550-base pair DNA segment and abnormal methylation in fragile X syndrome. Science.

[bib207] O’Dushlaine CT, Shields DC (2008). Marked variation in predicted and observed variability of tandem repeat loci across the human genome. BMC Genomics.

[bib208] Ogasawara M, Iida A, Kumutpongpanich T, Ozaki A, Oya Y, Konishi H, Nakamura A, Abe R, Takai H, Hanajima R, Doi H, Tanaka F, Nakamura H, Nonaka I, Wang Z, Hayashi S, Noguchi S, Nishino I (2020). Cgg expansion in NOTCH2NLC is associated with oculopharyngodistal myopathy with neurological manifestations. Acta Neuropathologica Communications.

[bib209] Oh SY, He F, Krans A, Frazer M, Taylor JP, Paulson HL, Todd PK (2015). Ran translation at CGG repeats induces ubiquitin proteasome system impairment in models of fragile X-associated tremor ataxia syndrome. Human Molecular Genetics.

[bib210] Okubo M, Doi H, Fukai R, Fujita A, Mitsuhashi S, Hashiguchi S, Kishida H, Ueda N, Morihara K, Ogasawara A, Kawamoto Y, Takahashi T, Takahashi K, Nakamura H, Kunii M, Tada M, Katsumoto A, Fukuda H, Mizuguchi T, Miyatake S, Miyake N, Suzuki J, Ito Y, Sone J, Sobue G, Takeuchi H, Matsumoto N, Tanaka F (2019). Ggc repeat expansion of NOTCH2NLC in adult patients with leukoencephalopathy. Annals of Neurology.

[bib211] Ordway JM, Tallaksen-Greene S, Gutekunst CA, Bernstein EM, Cearley JA, Wiener HW, Dure LS, Lindsey R, Hersch SM, Jope RS, Albin RL, Detloff PJ (1997). Ectopically expressed CAG repeats cause intranuclear inclusions and a progressive late onset neurological phenotype in the mouse. Cell.

[bib212] Orr HT (2012). Polyglutamine neurodegeneration: expanded glutamines enhance native functions. Current Opinion in Genetics & Development.

[bib213] Osadchuk L, Vasiliev G, Kleshchev M, Osadchuk A (2022). Androgen receptor gene CAG repeat length varies and affects semen quality in an ethnic-specific fashion in young men from russia. International Journal of Molecular Sciences.

[bib214] Otten AD, Tapscott SJ (1995). Triplet repeat expansion in myotonic dystrophy alters the adjacent chromatin structure. PNAS.

[bib215] Palazzolo I, Burnett BG, Young JE, Brenne PL, La Spada AR, Fischbeck KH, Howell BW, Pennuto M (2007). Akt blocks ligand binding and protects against expanded polyglutamine androgen receptor toxicity. Human Molecular Genetics.

[bib216] Pan B, Li R, Chen Y, Tang Q, Wu W, Chen L, Lu C, Pan F, Ding H, Xia Y, Hu L, Chen D, Sha J, Wang X (2016). Genetic association between androgen receptor gene CAG repeat length polymorphism and male infertility. Medicine.

[bib217] Pappalardo XG, Barra V (2021). Losing DNA methylation at repetitive elements and breaking bad. Epigenetics & Chromatin.

[bib218] Pascual M, Vicente M, Monferrer L, Artero R (2006). The muscleblind family of proteins: an emerging class of regulators of developmentally programmed alternative splicing. Differentiation; Research in Biological Diversity.

[bib219] Pastori C, Peschansky VJ, Barbouth D, Mehta A, Silva JP, Wahlestedt C (2014). Comprehensive analysis of the transcriptional landscape of the human FMR1 gene reveals two new long noncoding rnas differentially expressed in fragile X syndrome and fragile X-associated tremor/ataxia syndrome. Human Genetics.

[bib220] Patel PK, Bhavesh NS, Hosur RV (2000). Cation-Dependent conformational switches in d-TGGCGGC containing two triplet repeats of fragile X syndrome: NMR observations. Biochemical and Biophysical Research Communications.

[bib221] Paul S, Dansithong W, Jog SP, Holt I, Mittal S, Brook JD, Morris GE, Comai L, Reddy S (2011). Expanded CUG repeats Dysregulate RNA splicing by altering the stoichiometry of the muscleblind 1 complex. The Journal of Biological Chemistry.

[bib222] Paulson HL, Perez MK, Trottier Y, Trojanowski JQ, Subramony SH, Das SS, Vig P, Mandel JL, Fischbeck KH, Pittman RN (1997). Intranuclear inclusions of expanded polyglutamine protein in spinocerebellar ataxia type 3. Neuron.

[bib223] Paulson H, Geschwind DH, Paulson HL, Klein C (2018). Handbook of Clinical Neurology, Neurogenetics, Part I.

[bib224] Payseur BA, Jing P, Haasl RJ (2011). A genomic portrait of human microsatellite variation. Molecular Biology and Evolution.

[bib225] Philips AV, Timchenko LT, Cooper TA (1998). Disruption of splicing regulated by a CUG-binding protein in myotonic dystrophy. Science.

[bib226] Pieretti M, Zhang FP, Fu YH, Warren ST, Oostra BA, Caskey CT, Nelson DL (1991). Absence of expression of the FMR-1 gene in fragile X syndrome. Cell.

[bib227] Prado F, Piruat JI, Aguilera A (1997). Recombination between DNA repeats in yeast hpr1Delta cells is linked to transcription elongation. The EMBO Journal.

[bib228] Proops R, Webb T (1981). The “ fragile ” X chromosome in the martin-bell-renpenning syndrome and in males with other forms of familial mental retardation. Journal of Medical Genetics.

[bib229] Quartier A, Poquet H, Gilbert-Dussardier B, Rossi M, Casteleyn AS, Portes V, Feger C, Nourisson E, Kuentz P, Redin C, Thevenon J, Mosca-Boidron AL, Callier P, Muller J, Lesca G, Huet F, Geoffroy V, El Chehadeh S, Jung M, Trojak B, Le Gras S, Lehalle D, Jost B, Maury S, Masurel A, Edery P, Thauvin-Robinet C, Gérard B, Mandel JL, Faivre L, Piton A (2017). Intragenic FMR1 disease-causing variants: a significant mutational mechanism leading to fragile-X syndrome. European Journal of Human Genetics.

[bib230] Quilez J, Guilmatre A, Garg P, Highnam G, Gymrek M, Erlich Y, Joshi RS, Mittelman D, Sharp AJ (2016). Polymorphic tandem repeats within gene promoters act as modifiers of gene expression and DNA methylation in humans. Nucleic Acids Research.

[bib231] Qurashi A, Li W, Zhou JY, Peng J, Jin P (2011). Nuclear accumulation of stress response mRNAs contributes to the neurodegeneration caused by fragile X premutation rCGG repeats. PLOS Genetics.

[bib232] Ravikumar B, Vacher C, Berger Z, Davies JE, Luo S, Oroz LG, Scaravilli F, Easton DF, Duden R, O’Kane CJ, Rubinsztein DC (2004). Inhibition of mTOR induces autophagy and reduces toxicity of polyglutamine expansions in fly and mouse models of Huntington disease. Nature Genetics.

[bib233] Ravindran S (2012). Barbara mcclintock and the discovery of jumping genes. PNAS.

[bib234] Rodriguez CM, Todd PK (2019). New pathologic mechanisms in nucleotide repeat expansion disorders. Neurobiology of Disease.

[bib235] Rodriguez CM, Wright SE, Kearse MG, Haenfler JM, Flores BN, Liu Y, Ifrim MF, Glineburg MR, Krans A, Jafar-Nejad P, Sutton MA, Bassell GJ, Parent JM, Rigo F, Barmada SJ, Todd PK (2020). A native function for RAN translation and CGG repeats in regulating fragile X protein synthesis. Nature Neuroscience.

[bib236] Rosas I, Martínez C, Clarimón J, Lleó A, Illán-Gala I, Dols-Icardo O, Borroni B, Almeida MR, van der Zee J, Van Broeckhoven C, Bruni AC, Anfossi M, Bernardi L, Maletta R, Serpente M, Galimberti D, Scarpini E, Rossi G, Caroppo P, Benussi L, Ghidoni R, Binetti G, Nacmias B, Sorbi S, Piaceri I, Bagnoli S, Antonell A, Sánchez-Valle R, De la Casa-Fages B, Grandas F, Diez-Fairen M, Pastor P, Ferrari R, Álvarez V, Menéndez-González M (2020). Role for ATXN1, ATXN2, and HTT intermediate repeats in frontotemporal dementia and alzheimer’s disease. Neurobiology of Aging.

[bib237] Rubinsztein DC (2006). The roles of intracellular protein-degradation pathways in neurodegeneration. Nature.

[bib238] Sathasivam K, Neueder A, Gipson TA, Landles C, Benjamin AC, Bondulich MK, Smith DL, Faull RLM, Roos RAC, Howland D, Detloff PJ, Housman DE, Bates GP (2013). Aberrant splicing of HTT generates the pathogenic exon 1 protein in huntington disease. PNAS.

[bib239] Sato N, Amino T, Kobayashi K, Asakawa S, Ishiguro T, Tsunemi T, Takahashi M, Matsuura T, Flanigan KM, Iwasaki S, Ishino F, Saito Y, Murayama S, Yoshida M, Hashizume Y, Takahashi Y, Tsuji S, Shimizu N, Toda T, Ishikawa K, Mizusawa H (2009). Spinocerebellar ataxia type 31 is associated with “ inserted ” penta-nucleotide repeats containing (TGGAA) N. American Journal of Human Genetics.

[bib240] Schaefer MH, Wanker EE, Andrade-Navarro MA (2012). Evolution and function of CAG/polyglutamine repeats in protein-protein interaction networks. Nucleic Acids Research.

[bib241] Schilling G, Becher MW, Sharp AH, Jinnah HA, Duan K, Kotzuk JA, Slunt HH, Ratovitski T, Cooper JK, Jenkins NA, Copeland NG, Price DL, Ross CA, Borchelt DR (1999). Intranuclear inclusions and neuritic aggregates in transgenic mice expressing a mutant N-terminal fragment of huntingtin. Human Molecular Genetics.

[bib242] Schwartz M, Zlotorynski E, Kerem B (2006). The molecular basis of common and rare fragile sites. Cancer Letters.

[bib243] Sellier C, Rau F, Liu Y, Tassone F, Hukema RK, Gattoni R, Schneider A, Richard S, Willemsen R, Elliott DJ, Hagerman PJ, Charlet-Berguerand N (2010). Sam68 sequestration and partial loss of function are associated with splicing alterations in FXTAS patients. The EMBO Journal.

[bib244] Sellier C, Freyermuth F, Tabet R, Tran T, He F, Ruffenach F, Alunni V, Moine H, Thibault C, Page A, Tassone F, Willemsen R, Disney MD, Hagerman PJ, Todd PK, Charlet-Berguerand N (2013). Sequestration of Drosha and DGCR8 by expanded CGG RNA repeats alters microRNA processing in fragile X-associated tremor/ataxia syndrome. Cell Reports.

[bib245] Sellier C, Buijsen RAM, He F, Natla S, Jung L, Tropel P, Gaucherot A, Jacobs H, Meziane H, Vincent A, Champy M-F, Sorg T, Pavlovic G, Wattenhofer-Donze M, Birling M-C, Oulad-Abdelghani M, Eberling P, Ruffenach F, Joint M, Anheim M, Martinez-Cerdeno V, Tassone F, Willemsen R, Hukema RK, Viville S, Martinat C, Todd PK, Charlet-Berguerand N (2017). Translation of expanded CGG repeats into fmrpolyg is pathogenic and may contribute to fragile X tremor ataxia syndrome. Neuron.

[bib246] Sellier C, Cerro-Herreros E, Blatter M, Freyermuth F, Gaucherot A, Ruffenach F, Sarkar P, Puymirat J, Udd B, Day JW, Meola G, Bassez G, Fujimura H, Takahashi MP, Schoser B, Furling D, Artero R, Allain FHT, Llamusi B, Charlet-Berguerand N (2018). RbFOX1/MBNL1 competition for CCUG RNA repeats binding contributes to myotonic dystrophy type 1/type 2 differences. Nature Communications.

[bib247] Sharma M, Pandey GK (2015). Expansion and function of repeat domain proteins during stress and development in plants. Frontiers in Plant Science.

[bib248] Sherman SL, Morton NE, Jacobs PA, Turner G (1984). The marker (X) syndrome: a cytogenetic and genetic analysis. Annals of Human Genetics.

[bib249] Sherman SL, Jacobs PA, Morton NE, Froster-Iskenius U, Howard-Peebles PN, Nielsen KB, Partington MW, Sutherland GR, Turner G, Watson M (1985). Further segregation analysis of the fragile X syndrome with special reference to transmitting males. Human Genetics.

[bib250] Smith KP, Byron M, Johnson C, Xing Y, Lawrence JB (2007). Defining early steps in mRNA transport: mutant mRNA in myotonic dystrophy type I is blocked at entry into SC-35 domains. The Journal of Cell Biology.

[bib251] Sobczak K., Mezer M, Michlewski G, Krol J, Krzyzosiak WJ (2003). Rna structure of trinucleotide repeats associated with human neurological diseases. Nucleic Acids Research.

[bib252] Sobczak K, Michlewski G, de Mezer M, Kierzek E, Krol J, Olejniczak M, Kierzek R, Krzyzosiak WJ (2010). Structural diversity of triplet repeat rnas. Journal of Biological Chemistry.

[bib253] Sofola OA, Jin P, Qin Y, Duan R, Liu H, de Haro M, Nelson DL, Botas J (2007). Rna-Binding proteins hnRNP A2/B1 and CUGBP1 suppress fragile X CGG premutation repeat-induced neurodegeneration in a *Drosophila* model of FXTAS. Neuron.

[bib254] Solnick D, Lee SI (1987). Amount of RNA secondary structure required to induce an alternative splice. Molecular and Cellular Biology.

[bib255] Sone J, Mitsuhashi S, Fujita A, Mizuguchi T, Hamanaka K, Mori K, Koike H, Hashiguchi A, Takashima H, Sugiyama H, Kohno Y, Takiyama Y, Maeda K, Doi H, Koyano S, Takeuchi H, Kawamoto M, Kohara N, Ando T, Ieda T, Kita Y, Kokubun N, Tsuboi Y, Katoh K, Kino Y, Katsuno M, Iwasaki Y, Yoshida M, Tanaka F, Suzuki IK, Frith MC, Matsumoto N, Sobue G (2019). Long-Read sequencing identifies GGC repeat expansions in NOTCH2NLC associated with neuronal intranuclear inclusion disease. Nature Genetics.

[bib256] Soragni E, Petrosyan L, Rinkoski TA, Wieben ED, Baratz KH, Fautsch MP, Gottesfeld JM (2018). Repeat-associated non-ATG (ran) translation in fuchs’ endothelial corneal dystrophy. Investigative Opthalmology & Visual Science.

[bib257] Steinbach P, Gläser D, Vogel W, Wolf M, Schwemmle S (1998). The DMPK gene of severely affected myotonic dystrophy patients is hypermethylated proximal to the largely expanded CTG repeat. American Journal of Human Genetics.

[bib258] Strässer K, Masuda S, Mason P, Pfannstiel J, Oppizzi M, Rodriguez-Navarro S, Rondón AG, Aguilera A, Struhl K, Reed R, Hurt E (2002). Trex is a conserved complex coupling transcription with messenger RNA export. Nature.

[bib259] Subramanian PS, Nelson DL, Chinault AC (1996). Large domains of apparent delayed replication timing associated with triplet repeat expansion at FRAXA and FRAXE. American Journal of Human Genetics.

[bib260] Sulovari A, Li R, Audano PA, Porubsky D, Vollger MR, Logsdon GA, Warren WC, Pollen AA, Chaisson MJP, Eichler EE, Human Genome Structural Variation Consortium (2019). Human-specific tandem repeat expansion and differential gene expression during primate evolution. PNAS.

[bib261] Sun X, Li PP, Zhu S, Cohen R, Marque LO, Ross CA, Pulst SM, Chan HYE, Margolis RL, Rudnicki DD (2015). Nuclear retention of full-length HTT RNA is mediated by splicing factors MBNL1 and U2AF65. Scientific Reports.

[bib262] Sun JH, Zhou L, Emerson DJ, Phyo SA, Titus KR, Gong W, Gilgenast TG, Beagan JA, Davidson BL, Tassone F, Phillips-Cremins JE (2018). Disease-Associated short tandem repeats co-localize with chromatin domain boundaries. Cell.

[bib263] Sutcliffe JS, Nelson DL, Zhang F, Pieretti M, Caskey CT, Saxe D, Warren ST (1992). Dna methylation represses FMR-1 transcription in fragile X syndrome. Human Molecular Genetics.

[bib264] Tabet R, Schaeffer L, Freyermuth F, Jambeau M, Workman M, Lee C-Z, Lin C-C, Jiang J, Jansen-West K, Abou-Hamdan H, Désaubry L, Gendron T, Petrucelli L, Martin F, Lagier-Tourenne C (2018). CUG initiation and frameshifting enable production of dipeptide repeat proteins from ALS/FTD C9ORF72 transcripts. Nature Communications.

[bib265] Tacke R, Chen Y, Manley JL (1997). Sequence-specific RNA binding by an SR protein requires RS domain phosphorylation: creation of an srp40-specific splicing enhancer. PNAS.

[bib266] Tam V, Patel N, Turcotte M, Bossé Y, Paré G, Meyre D (2019). Benefits and limitations of genome-wide association studies. Nature Reviews. Genetics.

[bib267] Tassone F, Hagerman RJ, Loesch DZ, Lachiewicz A, Taylor AK, Hagerman PJ (2000a). Fragile X males with unmethylated, full mutation trinucleotide repeat expansions have elevated levels of FMR1 messenger RNA. American Journal of Medical Genetics.

[bib268] Tassone F, Hagerman RJ, Taylor AK, Gane LW, Godfrey TE, Hagerman PJ (2000b). Elevated levels of FMR1 mrna in carrier males: a new mechanism of involvement in the fragile-X syndrome. American Journal of Human Genetics.

[bib269] Tassone F, Iwahashi C, Hagerman PJ (2004). Fmr1 RNA within the intranuclear inclusions of fragile X-associated tremor/ataxia syndrome (FXTAS). RNA Biology.

[bib270] Tassone F, De Rubeis S, Carosi C, La Fata G, Serpa G, Raske C, Willemsen R, Hagerman PJ, Bagni C (2011). Differential usage of transcriptional start sites and polyadenylation sites in FMR1 premutation alleles. Nucleic Acids Research.

[bib271] Thomas JD, Sznajder ŁJ, Bardhi O, Aslam FN, Anastasiadis ZP, Scotti MM, Nishino I, Nakamori M, Wang ET, Swanson MS (2017). Disrupted prenatal RNA processing and myogenesis in congenital myotonic dystrophy. Genes & Development.

[bib272] Thomson JP, Skene PJ, Selfridge J, Clouaire T, Guy J, Webb S, Kerr ARW, Deaton A, Andrews R, James KD, Turner DJ, Illingworth R, Bird A (2010). Cpg islands influence chromatin structure via the CpG-binding protein cfp1. Nature.

[bib273] Tian Y, Wang J-L, Huang W, Zeng S, Jiao B, Liu Z, Chen Z, Li Y, Wang Y, Min H-X, Wang X-J, You Y, Zhang R-X, Chen X-Y, Yi F, Zhou Y-F, Long H-Y, Zhou C-J, Hou X, Wang J-P, Xie B, Liang F, Yang Z-Y, Sun Q-Y, Allen EG, Shafik AM, Kong HE, Guo J-F, Yan X-X, Hu Z-M, Xia K, Jiang H, Xu H-W, Duan R-H, Jin P, Tang B-S, Shen L (2019). Expansion of human-specific GGC repeat in neuronal intranuclear inclusion disease-related disorders. American Journal of Human Genetics.

[bib274] Todd PK, Oh SY, Krans A, Pandey UB, Di Prospero NA, Min K-T, Taylor JP, Paulson HL (2010). Histone deacetylases suppress CGG repeat-induced neurodegeneration via transcriptional silencing in models of fragile X tremor ataxia syndrome. PLOS Genetics.

[bib275] Todd PK, Oh SY, Krans A, He F, Sellier C, Frazer M, Renoux AJ, Chen K, Scaglione KM, Basrur V, Elenitoba-Johnson K, Vonsattel JP, Louis ED, Sutton MA, Taylor JP, Mills RE, Charlet-Berguerand N, Paulson HL (2013). Cgg repeat-associated translation mediates neurodegeneration in fragile X tremor ataxia syndrome. Neuron.

[bib276] Toulouse A, Au-Yeung F, Gaspar C, Roussel J, Dion P, Rouleau GA (2005). Ribosomal frameshifting on MJD-1 transcripts with long CAG tracts. Human Molecular Genetics.

[bib277] Trost B, Engchuan W, Nguyen CM, Thiruvahindrapuram B, Dolzhenko E, Backstrom I, Mirceta M, Mojarad BA, Yin Y, Dov A, Chandrakumar I, Prasolava T, Shum N, Hamdan O, Pellecchia G, Howe JL, Whitney J, Klee EW, Baheti S, Amaral DG, Anagnostou E, Elsabbagh M, Fernandez BA, Hoang N, Lewis MES, Liu X, Sjaarda C, Smith IM, Szatmari P, Zwaigenbaum L, Glazer D, Hartley D, Stewart AK, Eberle MA, Sato N, Pearson CE, Scherer SW, Yuen RKC (2020). Genome-Wide detection of tandem DNA repeats that are expanded in autism. Nature.

[bib278] Tsuchiya M, Nan H, Koh K, Ichinose Y, Gao L, Shimozono K, Hata T, Kim Y-J, Ohtsuka T, Cortese A, Takiyama Y (2020). Rfc1 repeat expansion in Japanese patients with late-onset cerebellar ataxia. Journal of Human Genetics.

[bib279] Tu M, Tong W, Perkins R, Valentine CR (2000). Predicted changes in pre-mRNA secondary structure vary in their association with exon skipping for mutations in exons 2, 4, and 8 of the HPRT gene and exon 51 of the fibrillin gene. Mutation Research/Mutation Research Genomics.

[bib280] Tuller T, Veksler-Lublinsky I, Gazit N, Kupiec M, Ruppin E, Ziv-Ukelson M (2011). Composite effects of gene determinants on the translation speed and density of ribosomes. Genome Biology.

[bib281] Udd B, Krahe R (2012). The myotonic dystrophies: molecular, clinical, and therapeutic challenges. The Lancet. Neurology.

[bib282] Uesaka M, Nishimura O, Go Y, Nakashima K, Agata K, Imamura T (2014). Bidirectional promoters are the major source of gene activation-associated non-coding RNAs in mammals. BMC Genomics.

[bib283] Uffelmann E, Huang QQ, Munung NS, de Vries J, Okada Y, Martin AR, Martin HC, Lappalainen T, Posthuma D (2021). Genome-wide association studies. Nature Reviews Methods Primers.

[bib284] Usdin K, Woodford KJ (1995). Cgg repeats associated with DNA instability and chromosome fragility form structures that block DNA synthesis in vitro. Nucleic Acids Research.

[bib285] Usdin K (2008). The biological effects of simple tandem repeats: lessons from the repeat expansion diseases. Genome Research.

[bib286] Usdin K, Kumari D (2015). Repeat-Mediated epigenetic dysregulation of the FMR1 gene in the fragile X-related disorders. Frontiers in Genetics.

[bib287] Verkerk AJ, Pieretti M, Sutcliffe JS, Fu YH, Kuhl DP, Pizzuti A, Reiner O, Richards S, Victoria MF, Zhang FP (1991). Identification of a gene (FMR-1) containing a CGG repeat coincident with a breakpoint cluster region exhibiting length variation in fragile X syndrome. Cell.

[bib288] Verma AK, Khan E, Mishra SK, Jain N, Kumar A (2019). Piperine modulates protein mediated toxicity in fragile X-associated tremor/ataxia syndrome through interacting expanded CGG repeat (R (CGG) EXP) RNA. ACS Chemical Neuroscience.

[bib289] Verma AK, Khan E, Mishra SK, Mishra A, Charlet-Berguerand N, Kumar A (2020). Curcumin regulates the R (CGG) EXP RNA hairpin structure and ameliorate defects in fragile X-associated tremor ataxia syndrome. Frontiers in Neuroscience.

[bib290] Verma AK, Khan E, Mishra SK, Kumar A (2022). Small molecule screening discovers compounds that reduce fmrpolyg protein aggregates and splicing defect toxicity in fragile X-associated tremor/ataxia syndrome. Molecular Neurobiology.

[bib291] Verstrepen KJ, Jansen A, Lewitter F, Fink GR (2005). Intragenic tandem repeats generate functional variability. Nature Genetics.

[bib292] Vinces MD, Legendre M, Caldara M, Hagihara M, Verstrepen KJ (2009). Unstable tandem repeats in promoters confer transcriptional evolvability. Science.

[bib293] Volle CB, Delaney S (2012). Cag/Ctg repeats alter the affinity for the histone core and the positioning of DNA in the nucleosome. Biochemistry.

[bib294] Voynov V, Verstrepen KJ, Jansen A, Runner VM, Buratowski S, Fink GR (2006). Genes with internal repeats require the THO complex for transcription. PNAS.

[bib295] Wang YH, Gellibolian R, Shimizu M, Wells RD, Griffith J (1996). Long CCG triplet repeat blocks exclude nucleosomes: a possible mechanism for the nature of fragile sites in chromosomes. Journal of Molecular Biology.

[bib296] Wang YH (2007). Chromatin structure of repeating CTG/CAG and CGG/CCG sequences in human disease. Frontiers in Bioscience.

[bib297] Wang ET, Cody NAL, Jog S, Biancolella M, Wang TT, Treacy DJ, Luo S, Schroth GP, Housman DE, Reddy S, Lécuyer E, Burge CB (2012). Transcriptome-Wide regulation of pre-mRNA splicing and mRNA localization by muscleblind proteins. Cell.

[bib298] Weinberg DE, Shah P, Eichhorn SW, Hussmann JA, Plotkin JB, Bartel DP (2016). Improved ribosome-footprint and mrna measurements provide insights into dynamics and regulation of yeast translation. Cell Reports.

[bib299] Willems T, Gymrek M, Highnam G, Mittelman D, Erlich Y, 1000 Genomes Project Consortium (2014). The landscape of human STR variation. Genome Research.

[bib300] Willems T, Gymrek M, Poznik GD, Tyler-Smith C, Erlich Y, 1000 Genomes Project Chromosome Y Group (2016). Population-Scale sequencing data enable precise estimates of Y-STR mutation rates. American Journal of Human Genetics.

[bib301] Willemsen R, Bontekoe CJM, Severijnen LA, Oostra BA (2002). Timing of the absence of FMR1 expression in full mutation chorionic villi. Human Genetics.

[bib302] Winchester CL, Ferrier RK, Sermoni A, Clark BJ, Johnson KJ (1999). Characterization of the expression of DMPK and SIX5 in the human eye and implications for pathogenesis in myotonic dystrophy. Human Molecular Genetics.

[bib303] Wojciechowska M, Olejniczak M, Galka-Marciniak P, Jazurek M, Krzyzosiak WJ (2014). Ran translation and frameshifting as translational challenges at simple repeats of human neurodegenerative disorders. Nucleic Acids Research.

[bib304] Wright SE, Rodriguez CM, Monroe J, Xing J, Krans A, Flores BN, Barsur V, Ivanova MI, Koutmou KS, Barmada SJ, Todd PK (2022). Cgg repeats trigger translational frameshifts that generate aggregation-prone chimeric proteins. Nucleic Acids Research.

[bib305] Wyner N, Barash M, McNevin D (2020). Forensic autosomal short tandem repeats and their potential association with phenotype. Frontiers in Genetics.

[bib306] Xi Z, Zinman L, Moreno D, Schymick J, Liang Y, Sato C, Zheng Y, Ghani M, Dib S, Keith J, Robertson J, Rogaeva E (2013). Hypermethylation of the CpG island near the G4C2 repeat in ALS with a C9orf72 expansion. American Journal of Human Genetics.

[bib307] Yamamoto A, Lucas JJ, Hen R (2000). Reversal of neuropathology and motor dysfunction in a conditional model of Huntington’s disease. Cell.

[bib308] Yap K, Mukhina S, Zhang G, Tan JSC, Ong HS, Makeyev EV (2018). A short tandem repeat-enriched RNA assembles a nuclear compartment to control alternative splicing and promote cell survival. Molecular Cell.

[bib309] Yin Q, Wang H, Li N, Ding Y, Xie Z, Jin L, Li Y, Wang Q, Liu X, Xu L, Li Q, Ma Y, Cheng Y, Wang K, Zhong C, Yu Q, Tang W, Chen W, Yang W, Zhang F, Ding C, Bao L, Zhou B, Hu P, Li J (2020). Dosage effect of multiple genes accounts for multisystem disorder of myotonic dystrophy type 1. Cell Research.

[bib310] Yu S, Pritchard M, Kremer E, Lynch M, Nancarrow J, Baker E, Holman K, Mulley JC, Warren ST, Schlessinger D (1991). Fragile X genotype characterized by an unstable region of DNA. Science.

[bib311] Zhang K, Donnelly CJ, Haeusler AR, Grima JC, Machamer JB, Steinwald P, Daley EL, Miller SJ, Cunningham KM, Vidensky S, Gupta S, Thomas MA, Hong I, Chiu S-L, Huganir RL, Ostrow LW, Matunis MJ, Wang J, Sattler R, Lloyd TE, Rothstein JD (2015). The C9orf72 repeat expansion disrupts nucleocytoplasmic transport. Nature.

[bib312] Zhang Y-J, Gendron TF, Grima JC, Sasaguri H, Jansen-West K, Xu Y-F, Katzman RB, Gass J, Murray ME, Shinohara M, Lin W-L, Garrett A, Stankowski JN, Daughrity L, Tong J, Perkerson EA, Yue M, Chew J, Castanedes-Casey M, Kurti A, Wang ZS, Liesinger AM, Baker JD, Jiang J, Lagier-Tourenne C, Edbauer D, Cleveland DW, Rademakers R, Boylan KB, Bu G, Link CD, Dickey CA, Rothstein JD, Dickson DW, Fryer JD, Petrucelli L (2016). C9Orf72 poly (GA) aggregates sequester and impair HR23 and nucleocytoplasmic transport proteins. Nature Neuroscience.

[bib313] Zu T, Gibbens B, Doty NS, Gomes-Pereira M, Huguet A, Stone MD, Margolis J, Peterson M, Markowski TW, Ingram MAC, Nan Z, Forster C, Low WC, Schoser B, Somia NV, Clark HB, Schmechel S, Bitterman PB, Gourdon G, Swanson MS, Moseley M, Ranum LPW (2011). Non-ATG–initiated translation directed by microsatellite expansions. PNAS.

[bib314] Zu T, Liu Y, Bañez-Coronel M, Reid T, Pletnikova O, Lewis J, Miller TM, Harms MB, Falchook AE, Subramony SH, Ostrow LW, Rothstein JD, Troncoso JC, Ranum LPW (2013). Ran proteins and RNA foci from antisense transcripts in c9orf72 ALS and frontotemporal dementia. PNAS.

[bib315] Zu T, Cleary JD, Liu Y, Bañez-Coronel M, Bubenik JL, Ayhan F, Ashizawa T, Xia G, Clark HB, Yachnis AT, Swanson MS, Ranum LPW (2017). Ran translation regulated by muscleblind proteins in myotonic dystrophy type 2. Neuron.

